# Detection of the KIAA1549-BRAF fusion gene in cells forming microvascular proliferations in pilocytic astrocytoma

**DOI:** 10.1371/journal.pone.0220146

**Published:** 2019-07-22

**Authors:** Shinji Yamashita, Hideo Takeshima, Fumitaka Matsumoto, Kouji Yamasaki, Tsuyoshi Fukushima, Hideyuki Sakoda, Masamitsu Nakazato, Kiyotaka Saito, Asako Mizuguchi, Takashi Watanabe, Hajime Ohta, Kiyotaka Yokogami

**Affiliations:** 1 Department of Neurosurgery, Division of Clinical Neuroscience, Faculty of Medicine, University of Miyazaki, Miyazaki, Japan; 2 Section of Oncopathology and Regenerative Biology, Department of Pathology, Faculty of Medicine, University of Miyazaki, Miyazaki, Japan; 3 Neurology, Respirology, Endocrinology and Metabolism, Department of Internal Medicine, Faculty of Medicine, University of Miyazaki, Miyazaki, Japan; University of Alabama at Birmingham, UNITED STATES

## Abstract

Microvascular proliferation (MVP), an aberrant vascular structure containing multilayered mitotically active endothelial- and smooth-muscle cells/pericytes, is a histopathological hallmark of glioblastoma multiforme (GBM). Although MVP tends to be associated with high-grade glioma, it has also been detected in WHO grade I pilocytic astrocytoma (PA). However, little is known about the mechanism underlying its formation. Using TP53 point mutations as a marker for tumor-derived cells, we earlier reported that MVP was partially converted from tumor cells via mesenchymal transition. In the current study we used the KIAA1549-BRAF fusion gene as a marker to assess whether MVPs in PA contained tumor-derived cells and/or phenotypically distinct tumor cells expressing vascular markers. cDNA synthesized from frozen tissue of six PA patients operated at our institute was analyzed to detect the KIAA1549-BRAF fusion gene by reverse transcription polymerase chain reaction (RT-PCR) assay. The breakpoint in the fusion gene was identified by long and accurate PCR (LA-PCR) and Sanger sequencing of genomic DNA. Distinct tumor cells and cellular components of MVP were obtained by laser microdissection. For the qualitative and quantitative detection of the KIAA1549-BRAF fusion gene we performed genomic and digital PCR assays. Fluorescence *in situ* hybridization (FISH) was used to assess gene fusion in cellular components of MVP. Samples from three PA patients harbored the KIAA1549 exon 15, BRAF exon 9 fusion gene. In two patient samples with abundant MVP, RT-PCR assay detected strong bands arising from the KIAA1549-BRAF fusion gene in both tumor cells and cellular components of MVP. Digital PCR showed that vis-à-vis tumor tissue, its relative expression in cellular components of MVP was 42% in one- and 76% in another sample. FISH revealed amplified signals in both tumor cells and cellular components of MVP indicative of tandem duplication. Our findings suggest that in patients with PA, some cellular components of MVP contained tumor derived cell and/or phenotypically distinct tumor cells expressing vascular markers.

## Introduction

Gliomas, the most common type of brain tumors, are characterized by their infiltrative growth and high vascularity. Among them, glioblastoma multiforme (GBM) is the most aggressive and lethal. It is most highly neovascularized and contains vascular structures, i.e. areas of microvascular proliferation (MVP), that are complex structures composed of both proliferating endothelial and smooth-muscle cells [1–4]. MVP is abundant in areas adjacent to necrosis and at the edge of tumor invasion where rapidly proliferating tumor cells increase the demand for oxygen [5]. Consequently, vascular endothelial growth factor A (VEGF-A), induced by HIF1α and hypoxia, is thought to be the most important mediator for assembling these special vascular structures [6, 7]. In randomized phase III trials of bevacizumab, a monoclonal antibody against human VEGF-A, the overall survival time of patients with newly-diagnosed glioblastoma was not significantly increased [8, 9]. Recent work indicated that vascular endothelial cells derived from tumor-initiating cells play an important role in resistance to anti-VEGF therapy [10].

Glioma stem-like cells (GSCs) can transdifferentiate into vascular endothelial cells [10–12]. It was shown that endothelial cells in GBM surgical samples carried the same genomic alteration as tumor cells, and GSCs characterized as CD133-positive could develop into endothelial cells. We also showed that cellular components of MVP in GBM harbored the same p53 mutation as surrounding tumor cells as analyzed by immunostaining and Sanger sequencing with the laser microdissection method [13]. These findings suggested that tumor neovascularization in GBM was partially achieved via endothelial differentiation of GSCs. Additionally, it has been reported that tumor derived endothelial cells showed resistance to anti-VEGF therapies [10–12]. As aberrant vascular structures and chemoresistant phenotypes may be strongly involved in gliomas, treatments that target tumor-derived endothelial cells may overcome their resistance to anti-VEGF therapies.

Although MVP is generally a hallmark of GBM and is characteristic in high-grade glioma, it was observed in pilocytic astrocytoma (PA), a grade I astrocytoma. PA is a circumscribed, slow-growing tumor that includes abundant MVP comprised of so-called ‘glomeruloid’ vessels that form complex structures composed of both proliferating endothelial- and smooth-muscle cells [4, 14, 15]. Although some studies showed that the expression of VEGF was similar in PA and GBM, and was considered to elicit MVP in PA, very little is known [16]. And same as GBM, MVP in PA is also considered as the candidate therapeutic target. Anti-VEGF therapies have been used to treat PA patients [17, 18]. In some adults with recurrent PA unresponsive to conventional therapy, bevacizumab was efficacious, however, in pediatric glioma patients, bevacizumab and irinotecan showed no effect [17, 18]. Resistance to anti-VEGF therapies must be overcome in PA patients. To develop anti-angiogenic strategies for their treatment, the mechanisms underlying neovascularization in PA must be identified.

In the present study, we investigated the possibility that MVPs in PA, as in GBM, harbor tumor derived endothelial cells and/or phenotypically distinct tumor cells expressing vascular markers. The KIAA1549-BRAF fusion gene, resulting from duplication of the BRAF gene at 7q34, is used as a marker of tumor derived cells and phenotypically distinct tumor cells expressing vascular markers; it is the most frequent genetic alteration in PA (>70%) [19]. To evaluate whether cellular components of MVP in PA harbor the KIAA1549-BRAF fusion, tumor samples from patients treated at our institution were analyzed by Sanger sequencing for breakpoints. With primers designed around the breakpoint, the presence of the KIAA1549-BRAF fusion in cellular components of MVP, strictly collected by laser microdissection methodology, were qualitatively and quantitatively evaluated by genomic and digital PCR, respectively. FISH analysis was also employed to detect KIAA1549-BRAF fusion.

## Materials and methods

### Patients and tumor samples

The Institutional Review Board of Miyazaki University Hospital, Japan, approved our study (approval number 0–0175). All patients provided written informed consent. We enrolled six newly diagnosed PA patients who had undergone surgery at our institution between November 2010 and February 2015. The general patient characteristics, including the age, sex, and tumor location, were obtained from medical records and are shown in Table 1. Tissue specimens (n = 6) obtained at surgery either underwent histological examination or were frozen in liquid nitrogen and stored at -80°C until processing for genomic DNA and total RNA extraction.

**Table 1:** Patient characteristics.

Patient	Age (years)	Sex	Tumor location
**S1**	**3**	**Female**	**Cerebellar vermis**
**S2**	**43**	**Male**	**Temporal**
**S3**	**11**	**Male**	**Occipital**
**S4**	**17**	**Female**	**Cerebellar hemisphere**
**S5**	**14**	**Male**	**Frontal**
**S6**	**5**	**Male**	**Basal ganglia~Temporal**

### cDNA synthesis and genomic DNA extraction

Total RNA was extracted from frozen tumor tissues using the Qiagen RNeasy Mini Kit (Qiagen, Valencia, CA, USA) according to the manufacturer’s instructions. Reverse transcription was with SuperScript VILO cDNA Synthesis Master Mix (Invitrogen, Grand Island, NY, USA) according to the manufacturer’s instructions in a thermal cycler at 25°C for 10 min, followed by 42°C for 60 min, 85°C for 5 min, and cooling to 4°C.

Genomic DNA was isolated from frozen tissues using the QIAamp DNA Mini Kit (Qiagen). To obtain micro-dissected samples we used paraffin-embedded sections and the QIAamp DNA Micro Kit (Qiagen) according to the manufacturer’s instructions. cDNA and genomic DNA samples were stored at -20°C until analysis.

### Collection of tumor and MVP constituent cells

Formalin-fixed, paraffin-embedded tissue was cut into 2-μm-thick sections, mounted on membrane slides (1.0 PEN NF, Carl Zeiss, Oberkochen, Germany), and stained with hematoxylin and eosin for the detection of MVP. We identified glomeruloid vessels as MVP. Tumor samples were micro-dissected with a laser under a PALM Micro Beam Laser Capture Microdissection microscope (Carl Zeiss); dissected samples were collected with Adhesive Cap 500 Clear (Carl Zeiss). Approximately 1,000–5,000 tumor cells and cellular components of MVP (30–40 MVPs) were collected for analysis.

### Polymerase chain reaction (PCR)

Reverse transcription PCR (RT-PCR) assays were performed in a total reaction volume of 25 μl containing 12.5 μl Go Taq HS (Promega, Madison, WI, USA), 9.5 μl distilled water, 1 μl of 10 μM of each primer, and 1 μl of cDNA- or genomic DNA samples. PCR amplification was in a GeneAmp PCR System 9700 (Applied Biosystems, Waltham, MA, USA). The thermal cycling conditions were: initial denaturation (95°C, 2 min), 35 cycles of denaturation (95°C, 30 sec), annealing (58°C, 30 sec), extension (72°C, 20 sec), final extension (72°C, 5 min). The PCR products were separated by electrophoresis on 2% agarose gels; the target bands were cut from the gels.

Long and accurate PCR (LA-PCR) assays were performed in a total reaction volume of 50 μl containing 10 x LA-PCR Buffer II (5 μl, Takara Bio Inc., Shiga, Japan), a dNTP mixture (8 μl), distilled water (35.5 μl), 1 μl of 10 μM of each primer, and 1 μl of a genomic DNA sample. PCR amplification was in a GeneAmp PCR System 9700 (Applied Biosystems). The thermal cycling conditions were: initial denaturation (94°C, 1 min), 30 cycles of denaturation (98°C, 10 sec), annealing (68°C, 20 min), final extension (72°C, 5 min). The PCR products were separated by electrophoresis on 0.9% agarose gels; the target bands were cut from the gels.

Digital PCR assays were on a QuantStudio 3D Digital PCR System (Thermo Fisher Scientific, Waltham, MA, USA). To 6 μl of genomic DNA we added 1.525 μl nuclease-free water, 7.25 μl QuantStudio 3D Digital PCR Master Mix, and 0.725 μl TaqMan Copy Number Reference Assay for detecting RNase P. To detect the KIAA-BRAF fusion gene we combined 5 μl of genomic DNA with 7.25 μl QuantStudio 3D Digital PCR Master Mix, 1.35 μl of each primer, and 0.375 μl of a TaqMan probe. Sample mixes were loaded onto chips and placed in the ProFlex 2x Flat PCR System (Thermo Fisher Scientific). The program was: stage 1 (96°C, 10 min), 40 cycles of stage 2a (54°C, 2 min), stage 2b (98°C, 30 sec), stage 3 (60°C, 2 min). The end-point fluorescence of the partitions on the chips was analyzed with the QuantStudio 3D Analysis Suite Cloud Software (Thermo Fisher Scientific). Table 2 shows the primer- and the TaqMan probe sequences.

**Table 2:** Primer sequences.

**Primers for screening with cDNA and LA-PCR**	
primer	Sequence
KIAA ex15 Fwd	ATAAAACGTTCTCCCAAGCCTCGCCGGAAACACCA
KIAA ex16 Fwd	AGCCGATGTGCAGACACCATCCTCGGTGGAACTGG
BRAF ex9 Rev	CCATCACCACGAAATCCTTGGTCTCTAATCAAGTC
**Primers for detecting Breakpoint**	
primer	Sequence
FP0	GAAACACCAGGTCAACGGCT
FP1	TAGGGGAGAGAGAACCAGCC
FP2	GTCCCTTCACTTCTCCAGCC
FP3	CAGCCAGCTGCTTGTTCTTG
FP4	GTTATAGCCGCTGGGGACTC
FP5	TCCTCACAGAGGGTATGGGG
FP6	TGGCTGTTTAGGGGCTTTCA
RP0	GACCAAGGATTTCGTGGTGA
RP1	TGGATGGATAAGGGCACACT
RP2	GAGGGATTGAATGGCCCCAA
RP3	GGCAAACACTACTAGCCCCA
RP4	GACTTAGGAAGAGCCAGCCC
RP5	CACCATTCCCCAAGCCTTCT
RP6	TGGACAGACTGAGAACAACCC
**Primers for Sanger sequencing of PCR amplicons**	
primer	Sequence
KIAA ex15 Fwd	GAAACACCAGGTCAACGGCT
BRAF ex9 Rev	GACCAAGGATTTCGTGGTGA
**Primers for LCM samples**	
primer	Sequence
S1 Fwd	CCTTATGCAACCAGCCATTT
S1 Rev	AACACAATGCATAGCCCACA
S6 Fwd	TGCCAAGAGAACCCAGAAAT
S6 Rev	CAAACCACTATGGAAGGCAAA
**Primers and Taqman probes for digital PCR**	
primer and Taqman	Sequence
S1B Fwd	GATCGCTTTAGATAGACCTTATGCAA
S1B Rev	ACAGTAAGTCACTAAAAGTTTTGGAACAA
S1B Taqman	ATTTCTTAGTTCATCTGTTCTC
S6B Fwd	TCTGCCAAGAGAACCCAGAAA
S6B Rev	CAAACACTACTAGCCCCAGGATTAAA
S6B Taqman	CTATCAATTAAAACCAGCTCTGT

### Sanger sequencing

To extract DNA fragments from gels for sequencing, the QIAquick Gel Extraction Kit (Qiagen) was used. Each product was sequenced on an ABI PRISM 310- or 3130xl Genetic Analyzer (Applied Biosystems). Sequencing reactions were performed using a Big Dye Terminator version 1.1 or version 3.1 Cycle Sequencing Kit (Applied Biosystems). The conditions were: initial denaturation (96°C, 1 min), 25 cycles of denaturation (30 sec), annealing (50°C, 5 sec), extension (60°C, 4 min). Sequence Scanner Software version 2 (Applied Biosystems) was used for sequence analysis.

### Detection of fusion breakpoints in genomic DNA

To detect fusion breakpoints, LA-PCR products were analyzed by Sanger sequencing. KIAA intron 15 and BRAF intron 8 that included breakpoints were approximately 7 kb in length. Using our seven sequencing primers located in each intron at 1,000-bp intervals, the area including breakpoints was identified by state-of-base calling; breakpoints were detected by detailed analysis of the base sequences (S1 Fig).

### Fluorescence in situ hybridization (FISH)

Dual-color interphase FISH was performed for KIAA-BRAF fusion gene detection. Probes that annealed the duplication site of BRAF was labeled with Texas red, and the centromere of chromosome 7q was labeled with fluorescein isothiocyanate. Both probes were purchased from GSP Laboratory Inc. (BRAF1/CEN7q Dual Color FISH Probe, product No. GC115, Kobe, Japan).

Sections were cut at a 2-μm thickness. After having been deparaffinized and boiled in pretreatment solution for 30 minutes, the slides were washed twice in 2 x saline-sodium citrate buffer (SSC) for 5 minutes. The slides were digested in protease solution at 37°C for 10–20 minutes, were rinsed twice with 2 x SSC, and were dehydrated with a 70%/100% ethanol series. The probes (10 μl) were applied, and the sections were covered with cover glasses and rubber cement. After having been denatured on a hot plate at 75°C for 5 minutes, the slides were incubated at 37°C for 26–50 hr in a humid chamber. After hybridization, the slides were washed with 2 x SSC/0.3% NP-40 for 5 minutes, 2 x SSC/0.3% NP-40 at 72°C for 1–2 minutes, and then 2 x SSC at room temperature for 5 minutes. The nuclei were counterstained with 4ʹ,6-diamidino-2-phenylindole, and signals were visualized by fluorescence microscopy.

### Immunohistochemistry

Serial sections (2 μm) were mounted on MAS-coated slides (Matsunami Glass Ind.,Ltd, Osaka, Japan) and processed for heat-induced antigen retrieval by autoclave with citrate buffer (pH 6.0) for OLIG2 or EDTA buffer (pH 9.0) for glial fibrillary acidic protein, CD34, and α-SMA, followed by treatment with 3% H_2_O_2_ in PBS for 10 minutes and were washed thrice in PBS. After blocking in 10% normal goat serum in PBS for 1 hour, the sections were incubated in moist chambers overnight at 4°C with antibodies to glial fibrillary acidic protein (rabbit polyclonal, prediluted; Dako Cytomation, Glostrup, Denmark), OLIG2 (rabbit polyclonal, dilution 1:100; IBL, Fujioka, Japan), CD34 (mouse monoclonal, dilution 1:50; Dako Cytomation), and α-SMA (mouse monoclonal, dilution 1:100; Dako Cytomation). After washing with PBS, the corresponding secondary antibody reaction was conducted. 3,3′-diaminobenzidine (DAB) solution was used for visualization. The slides were counterstained with hematoxylin, were dehydrated, and were cover-slipped.

### Statistics

The relative expression of the KIAA1549-BRAF fusion gene/RNase-P gene on digital PCR images was calculated using triplicate data from each sample (S1 and S6) analyzed with QuantStudio 3D Analysis Suite Cloud Software. Comparison between the samples (S1 and S6) was performed by Student’s t-test. Differences were considered significant at p<0.05. Statistical analyses were performed with Stat Mate III software (ATMS, Tokyo, Japan).

## Results

### Detection of the KIAA-BRAF fusion gene in patients with Pilocytic astrocytoma (PA)

**Fig 1:**
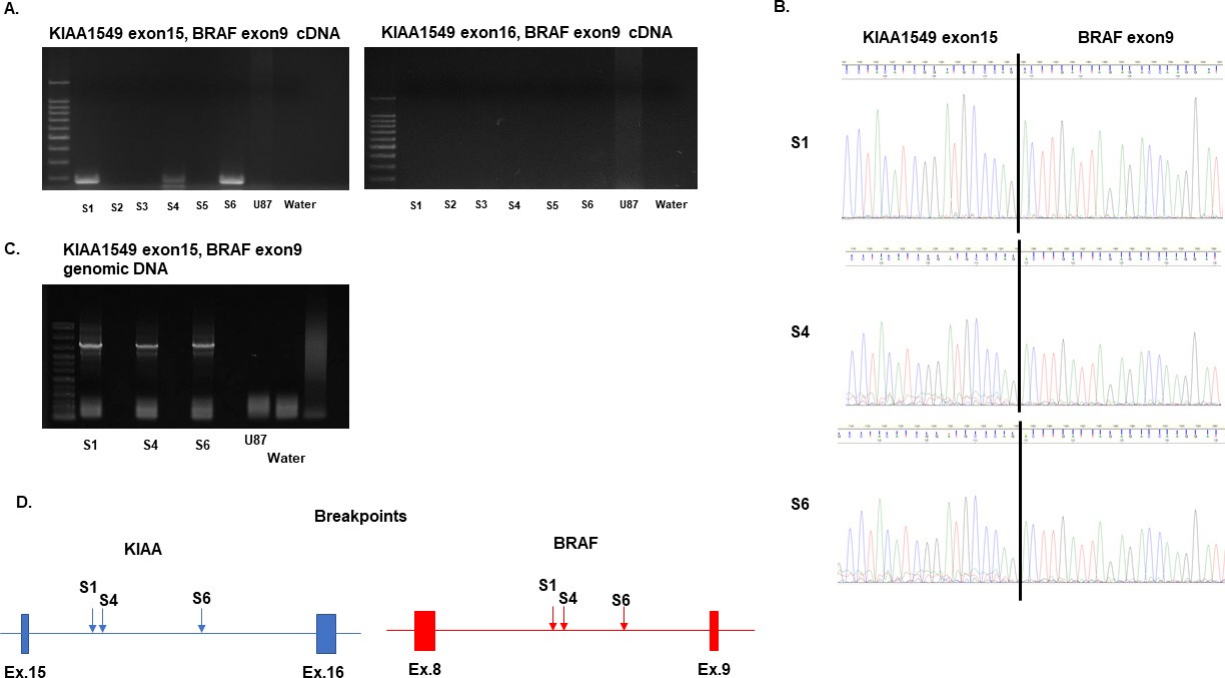
Screening for patient samples harboring the KIAA-BRAF fusion gene and breakpoint detection. (A) RT-PCR was performed to detect samples harboring the KIAA-BRAF fusion gene. cDNA of U87 and water were the negative controls. Among the six patient samples, S1, S4, and S6 expressed the KIAA1549 exon15, BRAF exon 9 fusion gene (left). None of the six samples manifested the KIAA1549 exon16, BRAF exon 9 fusion gene (right). (B) Sanger sequencing of S1, S4, and S6 revealed the KIAA1549 exon15, BRAF exon 9 fusion junctions. (C) LA-PCR assay of genomic DNA performed to obtain long amplified products including breakpoints. The genome of U87 and water were the negative controls. S1, S4, and S6 contained amplified products around 7 kb in length. (D) Breakpoints in S1, S4, and S6 detected by Sanger sequencing. Although the fusion pattern was the same, the breakpoints were different. For details, see S8 Fig.

To identify the KIAA-BRAF fusion gene in six patients with PA, we performed RT-PCR with synthesized cDNA from frozen tumor tissues. Table 1 shows the clinical characteristics of the 6 patients. PCR primers were used to assess frequent fusion patterns such as KIAA1549 exon 15, BRAF exon 9 and KIAA1549 exon 16, BRAF exon 9. KIAA1549 exon 15, BRAF exon 9 fusions were found in three samples (Fig 1A). Sanger sequencing verified KIAA1549 exon15, BRAF exon 9 fusion junctions in these samples (Fig 1B). In our series, no sample exhibited the KIAA1549 exon 16, BRAF exon 9 fusion pattern (Fig 1A).

### Determination of the breakpoints in the KIAA-BRAF fusion gene

To obtain the cellular components of MVP in PA, we used laser microdissection to collect cells from formalin-fixed, paraffin-embedded (FFPE) slides. However, as genomic DNA derived from FFPE slides is usually fragmented, we identified the accurate breakpoints of each KIAA1549-BRAF fusion gene.

In the three samples harboring the KIAA1549-BRAF fusion gene (S1, S4, S6), amplified products including breakpoints were obtained by long and accurate PCR (LA-PCR) with genomic DNA purified from frozen tissues (Fig 1C). Sanger sequencing using 7 sequencing primers at 1,000-bp intervals for each intron detected breakpoints in KIAA1549 intron 15 and BRAF intron 8 (Fig 1D and S2–S8 Figs). As in another report, each sample featured a distinct breakpoint despite exhibiting the same fusion pattern [20].

### Histological characteristics of MVP in PA

**Fig 2:**
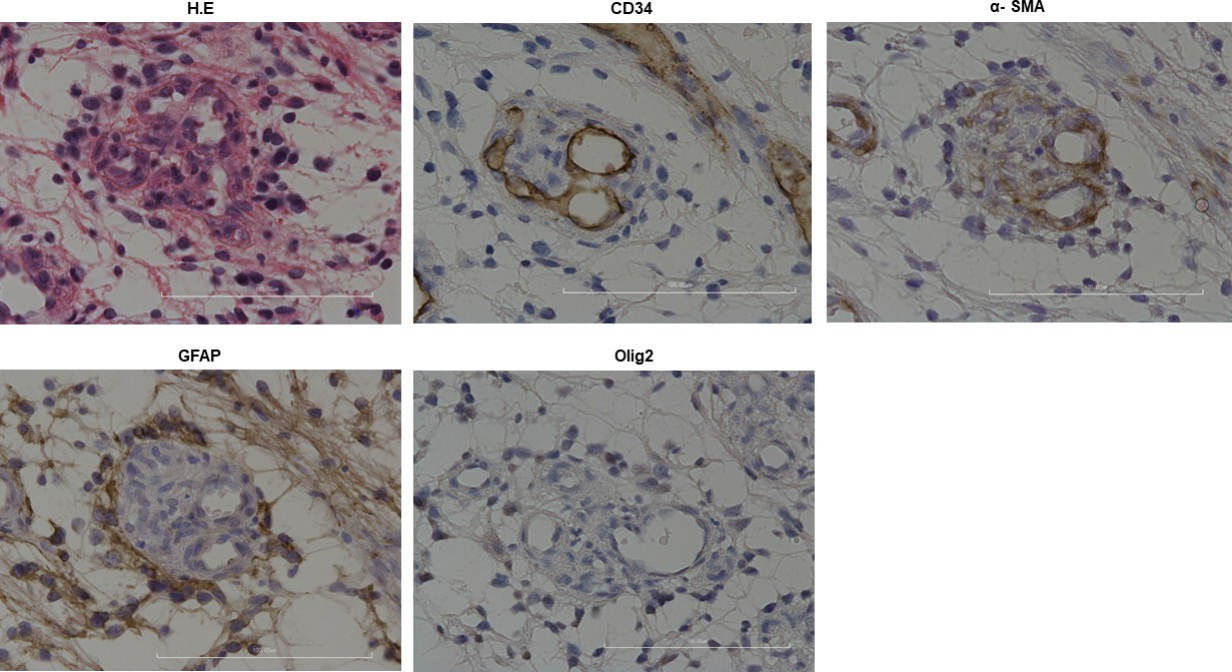
Histopathological findings of MVP in PA. H & E staining: MVP exhibiting a glomerular structure. CD34: Inner layer of MVP was stained against this marker. α-SMA: Cellular components of MVP were stained diffusely against this marker. GFAP and Olig2: No cellular components of MVP were stained against these markers. Original magnification: 20x. Scale bar: 100 μm.

To evaluate MVP in PA, we performed immunohistochemical studies. Neither of the tumor cell markers glial fibrillary acidic protein (GFAP) and Olig2 was detected. However, the vessel markers CD34 and α-smooth muscle actin (α-SMA) were observed (Fig 2). This means that none of MVP was directly invaded by surrounding original tumor cells.

### Detection of KIAA-BRAF fusion in MVP by PCR

**Fig 3:**
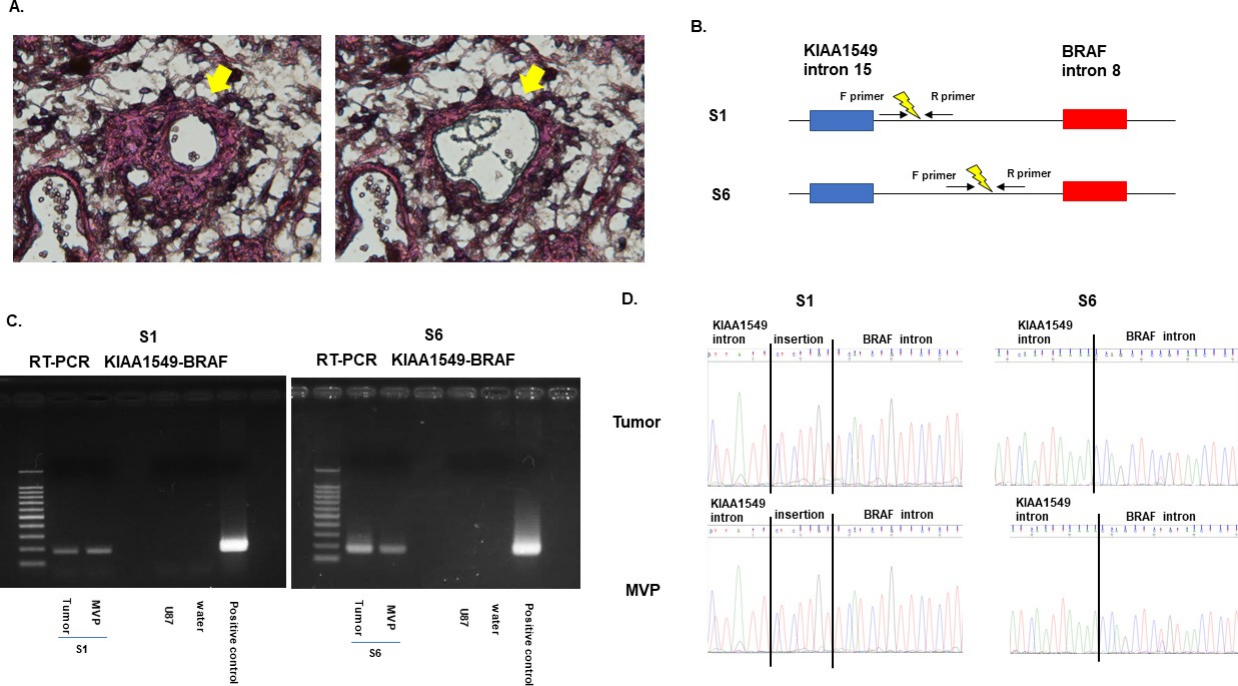
Evaluation of the KIAA-BRAF fusion gene in cellular components of MVP by genomic PCR. (A) Cellular components of MVP were collected from FFPE slides with laser microdissection (arrows). (B) Special primers designed for around breakpoints. (C) PCR analysis with the special primers was performed for fusion gene detection. Samples S1 and S6 exhibited a positive band for tumor and for cellular components of MVP. (D) Sanger sequencing was performed to confirm the amplified products. In S1 and S6, tumor cells and cellular components of MVP exhibited the same base sequence, including breakpoints.

**Fig 4:**
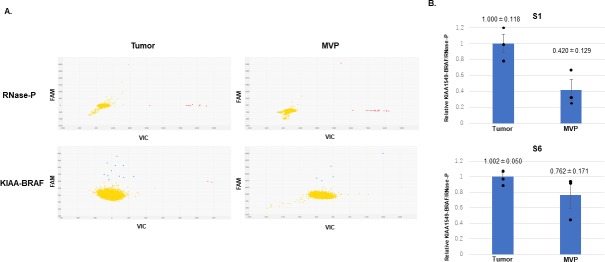
Digital PCR for quantitative fusion gene evaluation. (A) Raw digital PCR data for S1. RNase-P was employed as an internal control. Red dots showed droplets with RNase-P DNA and blue dots with the KIAA-BRAF fusion gene. Yellow dots showed droplets with no DNA. (B) The relative expression of the KIAA-BRAF fusion gene in tumor cells and cellular components of MVP is shown. The ratio of cells harboring the KIAA-BRAF fusion gene (cellular components of MVP vs. tumor cells) was 42% in S1 and 76% in S6. The results are expressed as the mean +- S.E.M., n = 3.

To determine whether cellular components of MVP and tumor cells in PA harbored the same KIAA1549-BRAF fusion gene, we evaluated cells collected from FFPE slides by laser microdissection (Fig 3A and S9 Fig). Two samples (S1 and S6) with abundant MVP were selected for analysis. In both samples, PCR yielded bands for the KIAA1549-BRAF fusion gene in tumor cells and cellular components of MVP (Fig 3B and 3C). PCR analysis was performed in triplicate to ascertain reproducibility (data not shown). Sanger sequencing confirmed the expected breakpoints in the amplified products (Fig 3D). These data suggest that some cellular components of MVP contained tumor derived cell and/or phenotypically distinct tumor cells expressing vascular markers. Additionally, to further analyze the ratio of cells harboring the KIAA1549-BRAF fusion gene in cellular components of MVP compared to tumor cells, digital PCR with customized primers and Taqman probes was carried out. The results showed that the ratio of cells harboring the KIAA1549-BRAF fusion was 42% in cellular components of MVP compared to tumor cells in S1 and 76% in S6 (Fig 4 and S10 Fig). There was no significant difference in the ratio of cells harboring the KIAA1549-BRAF fusion gene in cellular components of MVP compared to tumor cells between S1 and S6 (p = 0.096).

### Detection of KIAA-BRAF fusion in MVP by FISH

**Fig 5:**
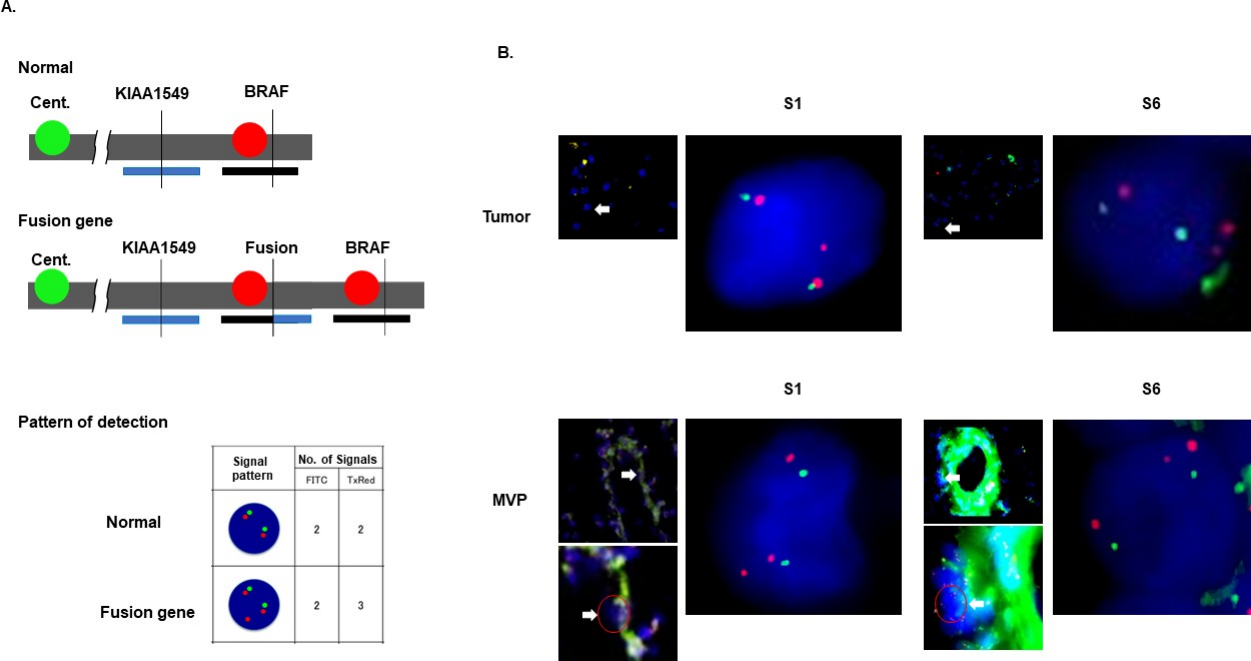
FISH analysis for the detection of the KIAA-BRAF fusion gene. (A) FISH probes at the duplication site of the BRAF gene (7q34) are labeled with Texas red. The centromere of chromosome 7q (7q11.21) is labeled with fluorescein isothiocyanate (FITC, green). Due to tandem duplication at 7q34 leading to the expression of the KIAA-BRAF fusion gene, cells with the fusion gene demonstrated gain of a red signal (total: three signals) in comparison with the green signal (two signals). (B) In both S1 and S6, tumor cells and cellular components of MVP were positive for the KIAA-BRAF fusion gene.

FISH was used to visually evaluate the KIAA1549-BRAF fusion in MVP. In PA, tandem duplication at 7q34 results in such fusion. Therefore, the combination signal pattern of BRAF-specific probes placed on the duplicated site (red) and on the centromere of chromosome 7 (green) detected the KIAA1549-BRAF fusion gene (Fig 5A). Not only tumor cells but also some cellular components of MVP exhibited a positive signal pattern for the fusion gene (Fig 5B). These findings also demonstrated that MVP in PA contained tumor derived cell and/or phenotypically distinct tumor cells expressing vascular markers.

## Discussion

New insight of the mechanism of GBM neovascularization that GSCs could transdifferentiate into vascular endothelial cells was much impressive not only for one of the mechanisms of the resistance of anti- VEGF therapies but for the interaction of GSCs with vascular compartments took part in the microenvironment [10–12, 20]. By contrast, little is known about neovascularization, vascular niche, and the existence of GSCs in PA. In this study, we evaluated whether vascular endothelial cells of PA contained tumor derived cells and/or phenotypically distinct tumor cells expressing vascular markers. This is the first report considering the origin of MVP in PA, and our results might shed light on the microenvironment in low-grade glioma.

To analyze samples harvested by laser microdissection from FFPE slides, we first identified the accurate breakpoint in each sample. Although three fusion-positive samples exhibited the same fusion pattern (KIAA1549 exon 15, BRAF exon 9), the breakpoints were different. Lawson et al. also reported that the breakpoint of each samples was totally different in their analysis of 43 low-grade astrocytoma patients, and these fusions could be categorized into three groups: “seamless transition,” “presence of short inserted,” and “breakpoint microhomology.” This terminology is based on the sequence alignment of the fusion breakpoint [21]. In our fusion positive samples, S6 showed a continuous transition from KIAA1549 intron 15 to BRAF intron 8 and, thus, was categorized as “seamless transition” (S3 Fig), S1 showed the 7 bp inserted sequence at the breakpoint and categorized as “presence of short inserted” (S5 Fig), and S4 showed the shared 3 bp sequence between the end of KIAA1549 and the beginning of BRAF and categorized as “breakpoint microhomology” (S7 Fig). We think that these results document the accuracy of our method for detecting breakpoints.

Our result showed that the ratio of cells harboring the KIAA1549-BRAF fusion gene in cellular components of MVP to tumor cells was different from S1 (42%) to S6 (76%). Soda et al. also showed a difference among GBM samples in the rate of endothelial cells derived from tumor cells (2.0–37.8%) [10]. They suggested that the observed rate variation was attributable to the location of the analyzed endothelial cells in the tumor, or to the tumor size *per se*. At present, we cannot rule out sampling bias due to the heterogeneity of our tumor cells and of the cellular components of MVP. We are collecting more samples to identify factors responsible for the inter-sample difference in the rate of cellular components of MVP that harbor the KIAA1549-BRAF fusion gene in patients with PA.

In this study, we demonstrated that MVP in PA contained tumor derived cell and/or phenotypically distinct tumor cells expressing vascular markers by analyzing the presence of the KIAA-BRAF fusion gene via PCR and FISH methods. In GBM, Vitiani et al. demonstrated that GSCs cultured in endothelial condition medium could generate progeny with the phenotypic and functional features of endothelial cells, resulting in tube formation and CD31 expression. GSCs injected into mice produced tumor xenografts, the vessels of which were composed primarily of human endothelial cells [11]. However, in PA, it is difficult to perform such in vitro and in vivo experiments because there are currently no methods for selecting and culturing GSCs from these tumors. Additionally, we also have no commercially available cell lines of PA. Thus, it will be critical to establish such methods to handle GSCs in PA and perform such verification experiments in the future.

## Conclusions

We provide evidence that MVP in PA might contain tumor derived cell and/or phenotypically distinct tumor cells expressing vascular markers. Our work sheds light on not only the resistance of PA to anti-VEGF therapies but also on the microenvironment in low-grade glioma.

## Supporting information

S1 Fig The design of sequence primers for the detection of KIAA1549/BRAF breakpoints. Sequencing primers (7 each) to be located at KIAA and BRAF introns at 1,000-bp intervals were designed to detect breakpoints.
(TIF)

S2 Fig Sequencing the genomic amplicon from LA-PCR of S6. S6 amplicons were subjected to Sanger sequencing. Red circles indicate that the base sequence annealed with the sequence primer. State-of-base calling identified areas of interest in the intron that included the breakpoint. In S6 the breakpoint was located between primers FP4 and RP3.
(TIF)

S3 Fig The KIAA1549-BRAF RP3 sequence in S6. The breakpoint in S6 was detected by Sanger sequencing using the RP3 primer. The fusion was categorized as a “seamless transition” from KIAA intron 15 to BRAF intron 8.
(TIF)

S4 Fig Sequencing the genomic amplicon from LA-PCR of S1. S1 amplicons were subjected to Sanger sequencing. The breakpoint was located between the FP1 and the RP5 primers.
(TIF)

S5 Fig The KIAA1549-BRAF FP1 sequence in S1. The breakpoint in S1 was detected by Sanger sequencing using the FP1 primer. The fusion was categorized as “presence of short insert”. A 7-bp sequence was inserted at the breakpoint.
(TIF)

S6 Fig RT-PCR with the KIAA1549 FP1-BRAF RP5 primer set. PCR analysis with FP1 and RP5 primers revealed bands in S1 and S4. As in S1 (S4 Fig.), the breakpoint in S4 was located between primers FP1 and RP5.
(TIF)

S7 Fig The KIAA1549-BRAF RP5 sequence in S4. The RP5 primer was used. The breakpoint in S4 was detected by sequencing. This fusion was categorized as “breakpoint microhomology”. We observed a shared 3-bp sequence between the end of KIAA and the start of BRAF.
(TIF)

S8 Fig Breakpoint locations in S1, S4, and S6. The location of breakpoints between KIAA intron 15 and BRAF intron 8 in S1, S4, and S6 is shown. Note the inter-sample difference in the breakpoint. S1: Magenta: KIAA intron 15, Purple: BRAF intron 8. S4: Aqua: KIAA intron 15, Green: BRAF intron 8. S6: Red: KIAA intron 15, Blue: BRAF intron 8.
(TIF)

S9 Fig Collection site of tumor cells and the cellular component of MVP. Tumor samples were collected from tumor tissue without MVP. Tumor samples and the cellular components of MVP were collected from the same tissue.
(TIF)

S10 Fig Result of digital PCR assay of S6. Raw digital PCR data for S6 are shown. Samples from tumor cells and cellular MVP components were independently analyzed. Top: Red and yellow dots indicate droplets with RNase-P DNA and droplets with no DNA, respectively. Bottom: Green and red dots identify droplets with the KIAA-BRAF fusion gene and droplets with no DNA, respectively.
(TIF)

S1 Dataset Raw data of figures in this manuscript. Raw data of Figs 1B, 3D, 4A, 4B, 5 and S3, S5, S7, S8 and S9 Figs are shown by power point or excel files. Original sequencing data and digital PCR data could be seen with adequate software (Sequence scanner version 2 and QuantStudio 3D Analysis Suite Cloud). These raw data are also available at Dryad digital repository (DOI: https://doi.org/10.5061/dryad.bv44rk5).
(ZIP)
