# Supplementary figures and images for: Detection of the KIAA1549-BRAF fusion gene in cells forming microvascular proliferations in pilocytic astrocytoma
Source: PLoS One. 2019 Jul 22;14(7):e0220146. doi: 10.1371/journal.pone.0220146 (PMC6645544; doi:10.1371/journal.pone.0220146)

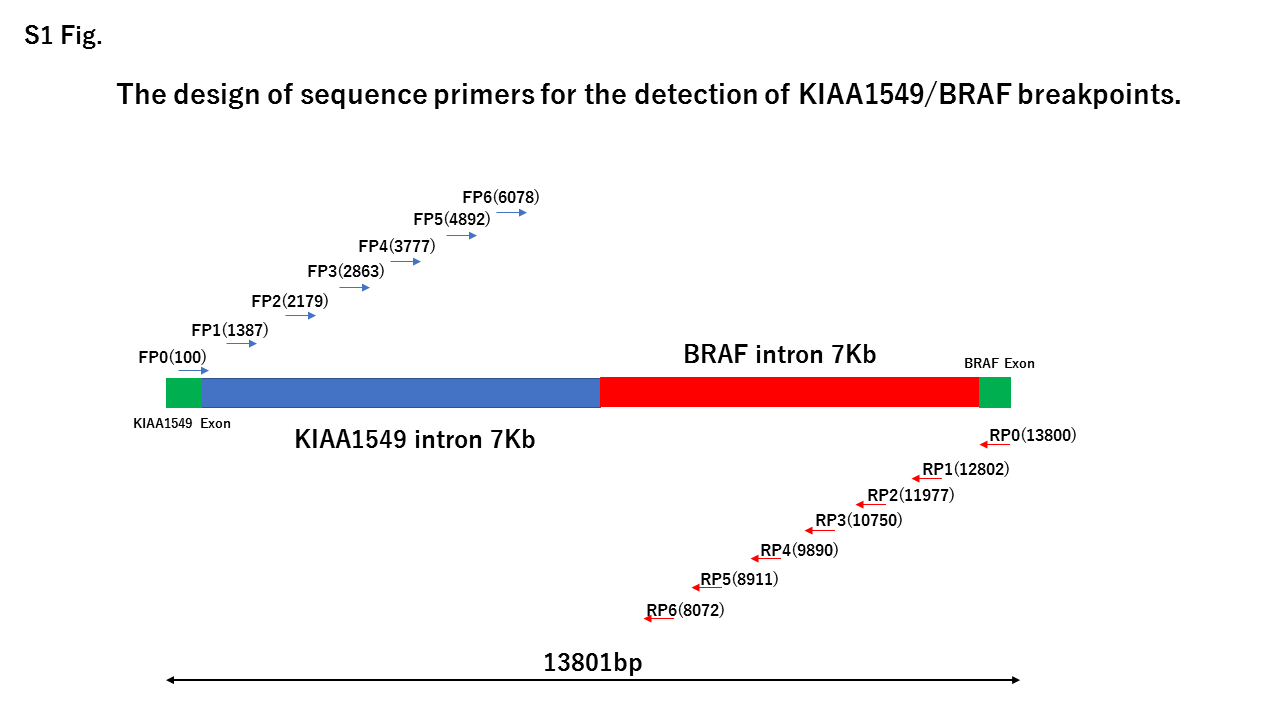

Supplement: S1 Fig — Sequencing primers (7 each) to be located at KIAA and BRAF introns at 1,000-bp intervals were designed to detect breakpoints. (TIF) [file pone.0220146.s001.tif]

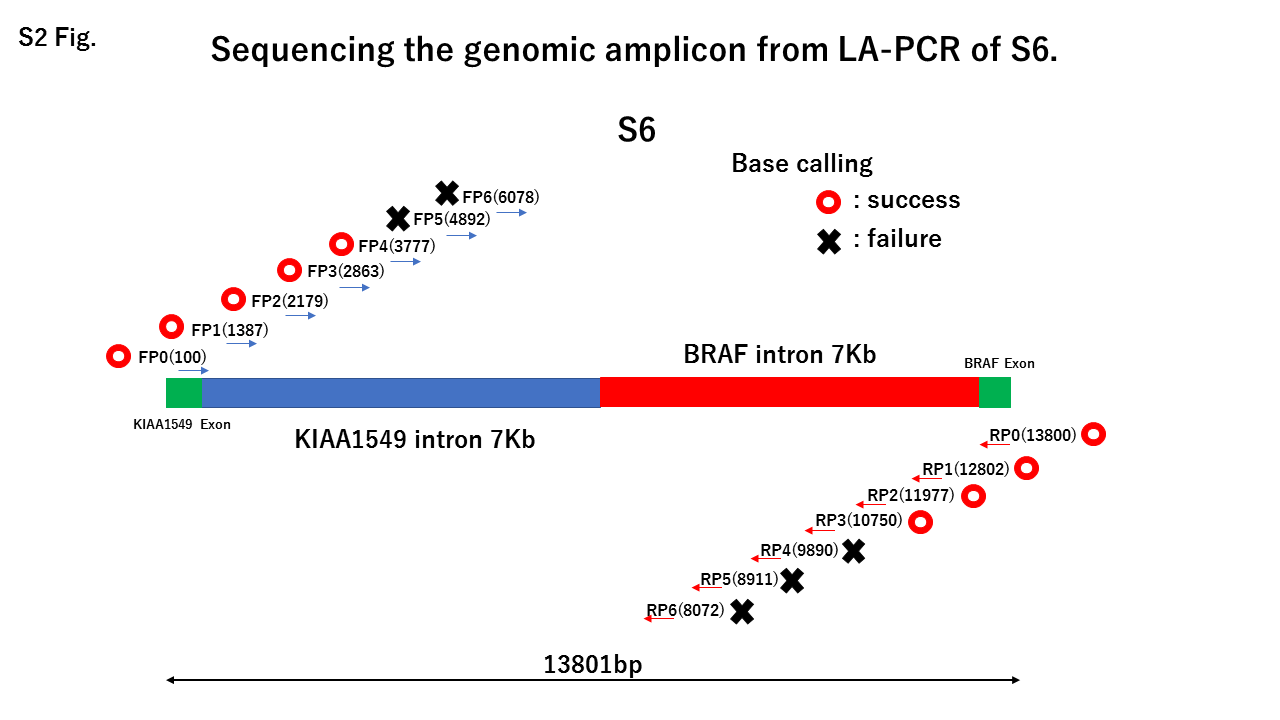

Supplement: S2 Fig — S6 amplicons were subjected to Sanger sequencing. Red circles indicate that the base sequence annealed with the sequence primer. State-of-base calling identified areas of interest in the intron that included the breakpoint. In S6 the breakpoint was located between primers FP4 and RP3. (TIF) [file pone.0220146.s002.tif]

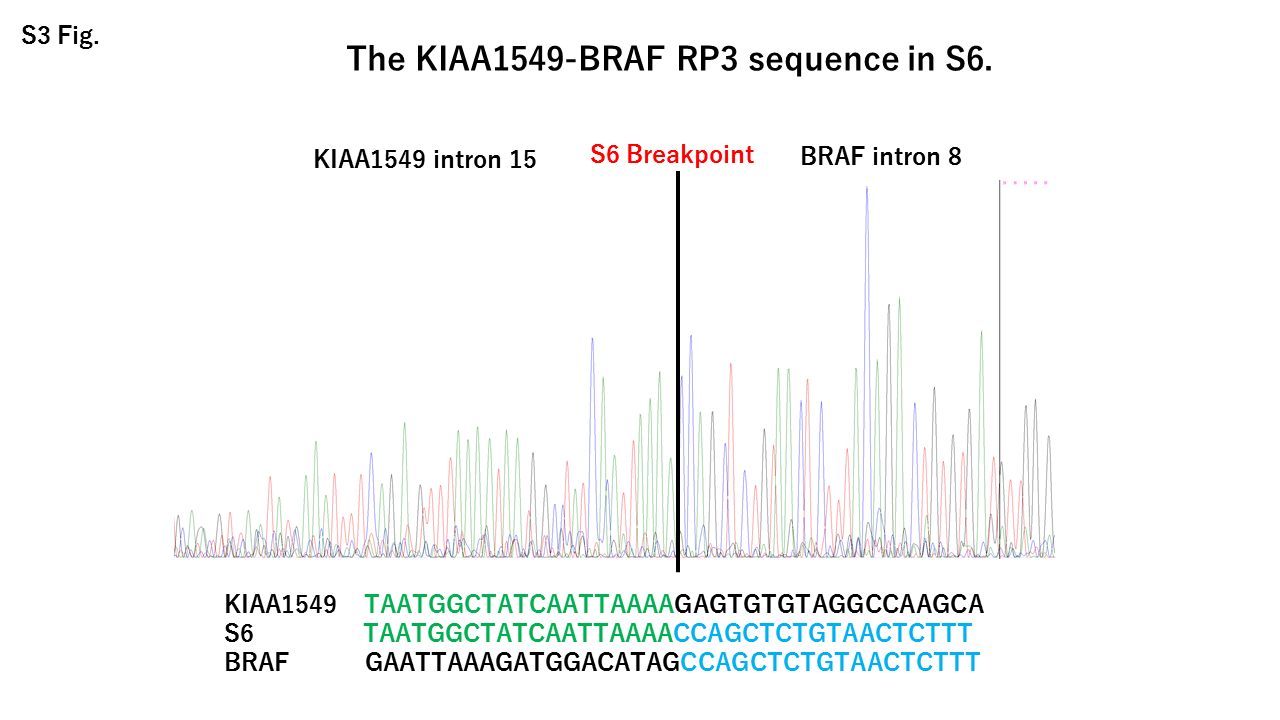

Supplement: S3 Fig — The breakpoint in S6 was detected by Sanger sequencing using the RP3 primer. The fusion was categorized as a “seamless transition” from KIAA intron 15 to BRAF intron 8. (TIF) [file pone.0220146.s003.tif]

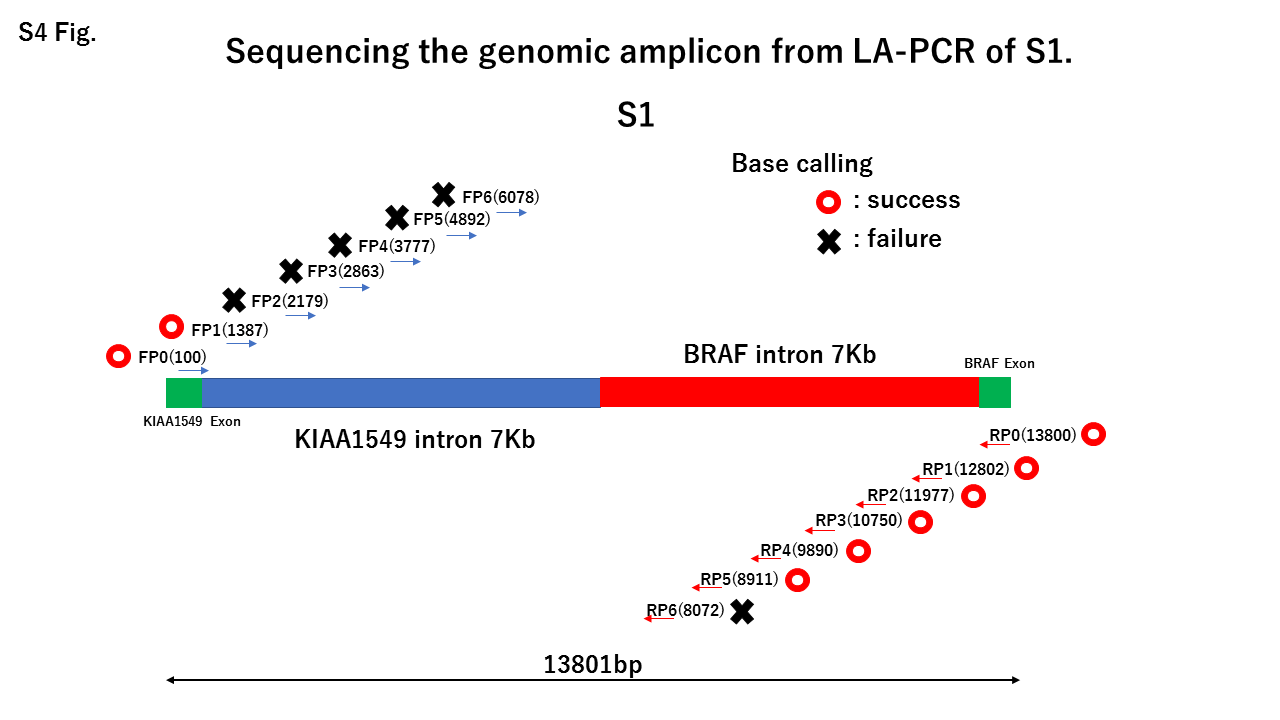

Supplement: S4 Fig — S1 amplicons were subjected to Sanger sequencing. The breakpoint was located between the FP1 and the RP5 primers. (TIF) [file pone.0220146.s004.tif]

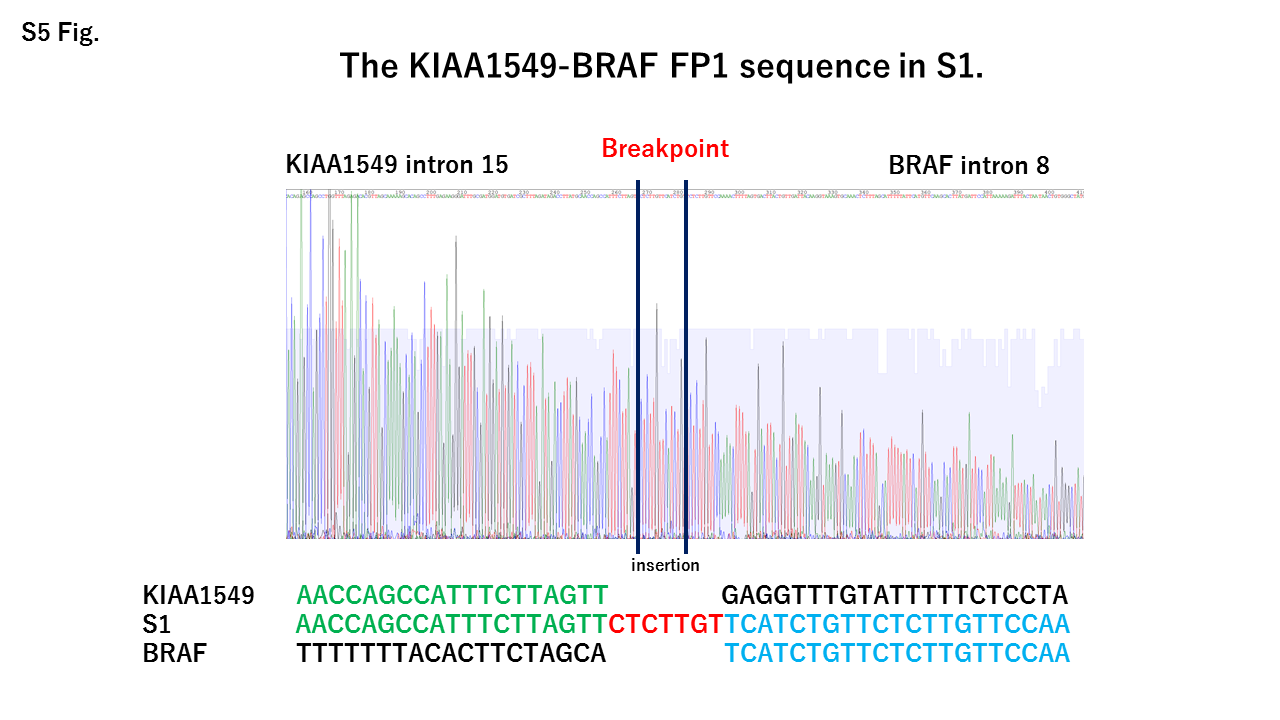

Supplement: S5 Fig — The breakpoint in S1 was detected by Sanger sequencing using the FP1 primer. The fusion was categorized as “presence of short insert”. A 7-bp sequence was inserted at the breakpoint. (TIF) [file pone.0220146.s005.tif]

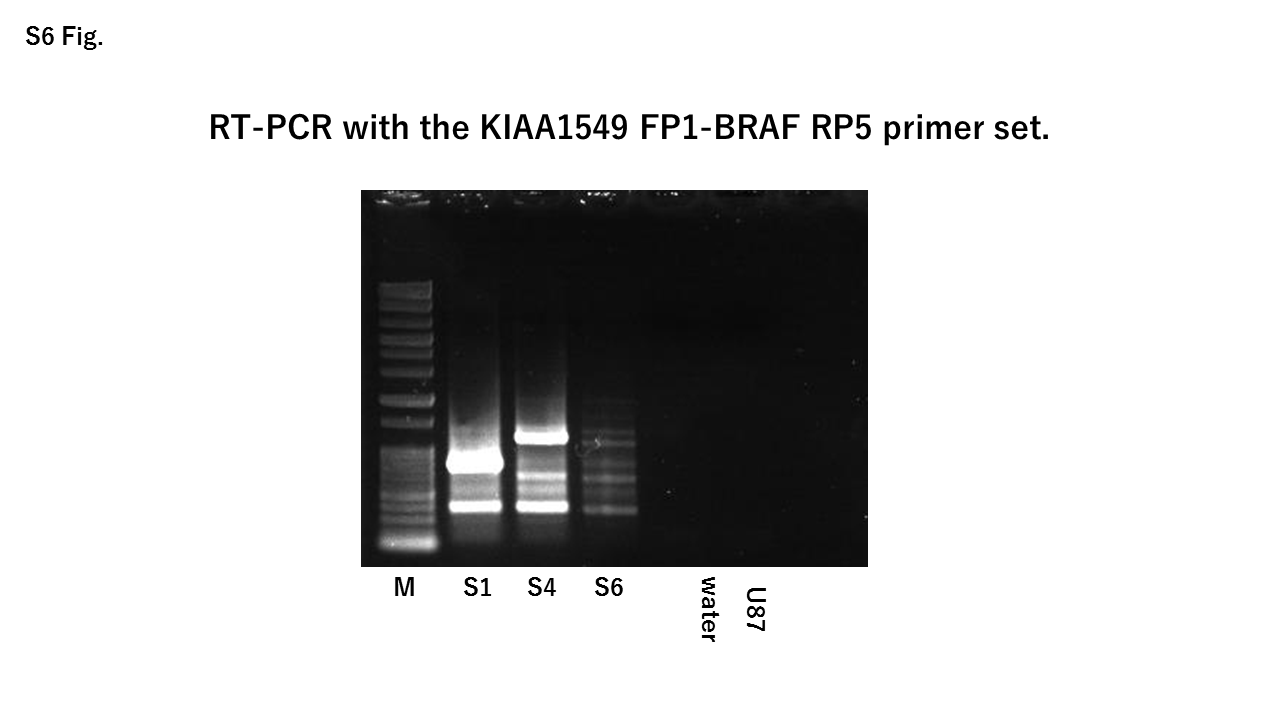

Supplement: S6 Fig — PCR analysis with FP1 and RP5 primers revealed bands in S1 and S4. As in S1 (S4 Fig.), the breakpoint in S4 was located between primers FP1 and RP5. (TIF) [file pone.0220146.s006.tif]

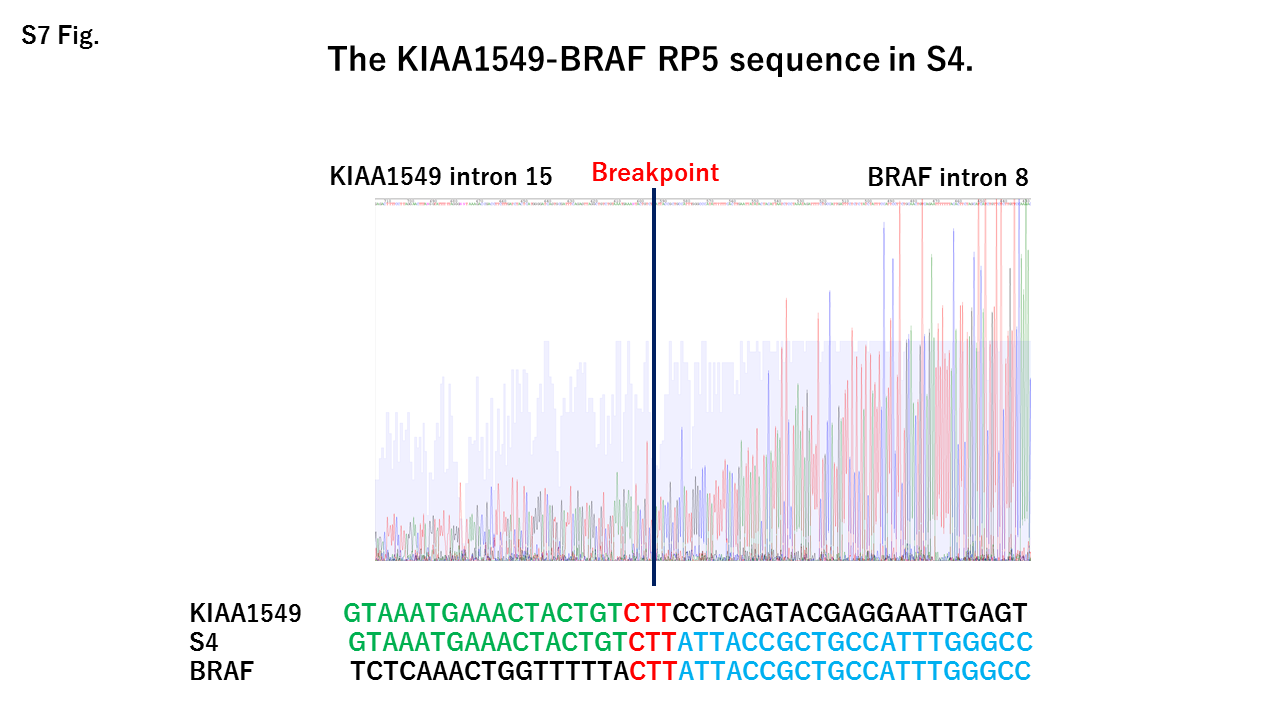

Supplement: S7 Fig — The RP5 primer was used. The breakpoint in S4 was detected by sequencing. This fusion was categorized as “breakpoint microhomology”. We observed a shared 3-bp sequence between the end of KIAA and the start of BRAF. (TIF) [file pone.0220146.s007.tif]

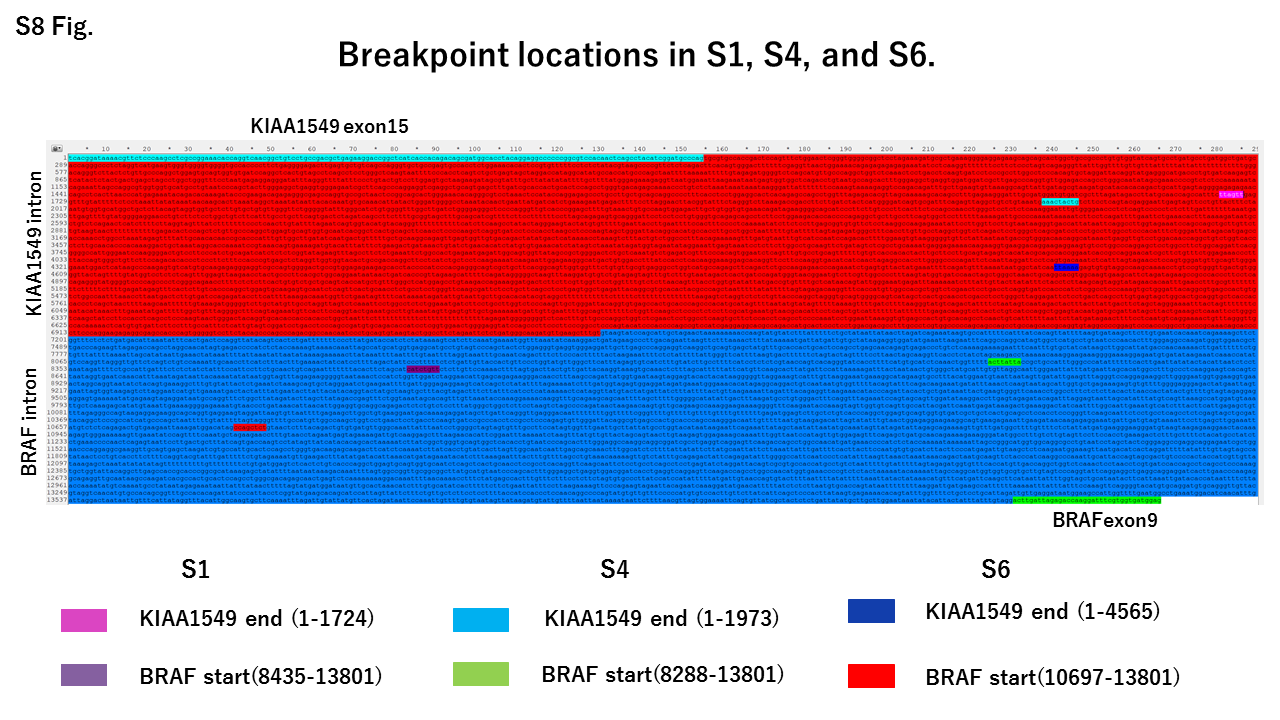

Supplement: S8 Fig — The location of breakpoints between KIAA intron 15 and BRAF intron 8 in S1, S4, and S6 is shown. Note the inter-sample difference in the breakpoint. S1: Magenta: KIAA intron 15, Purple: BRAF intron 8. S4: Aqua: KIAA intron 15, Green: BRAF intron 8. S6: Red: KIAA intron 15, Blue: BRAF intron 8. (TIF) [file pone.0220146.s008.tif]

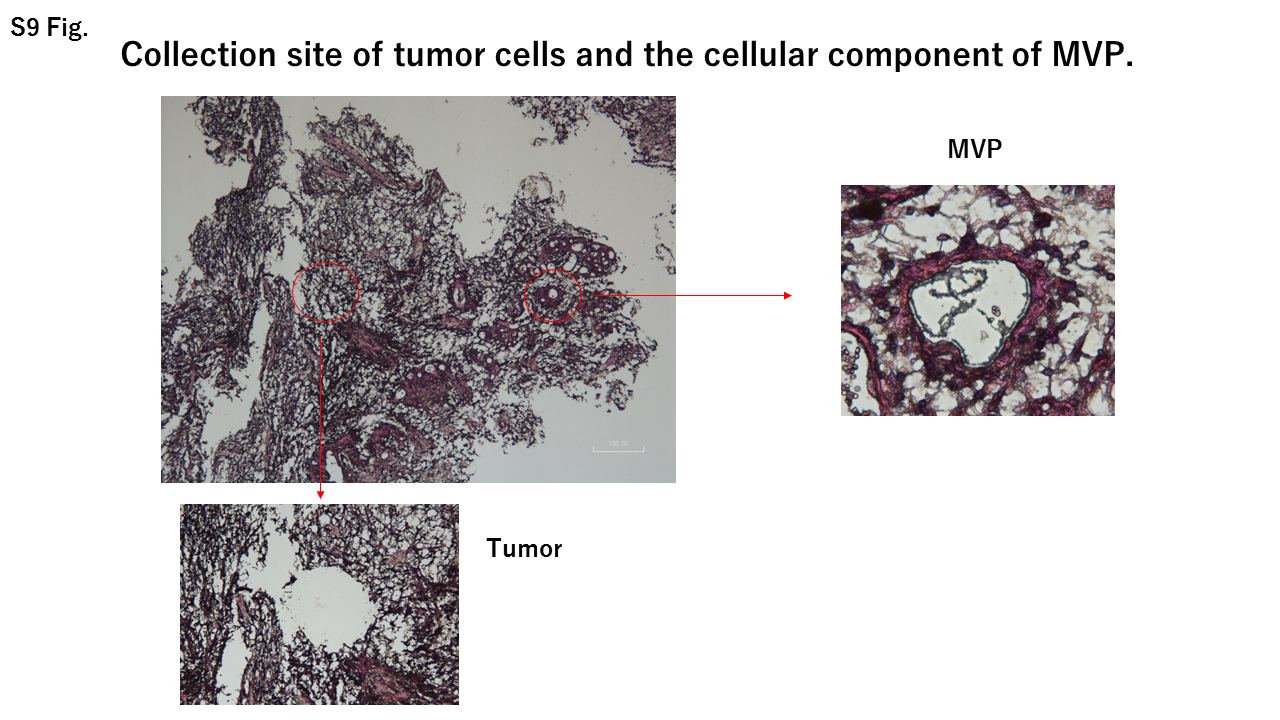

Supplement: S9 Fig — Tumor samples were collected from tumor tissue without MVP. Tumor samples and the cellular components of MVP were collected from the same tissue. (TIF) [file pone.0220146.s009.tif]

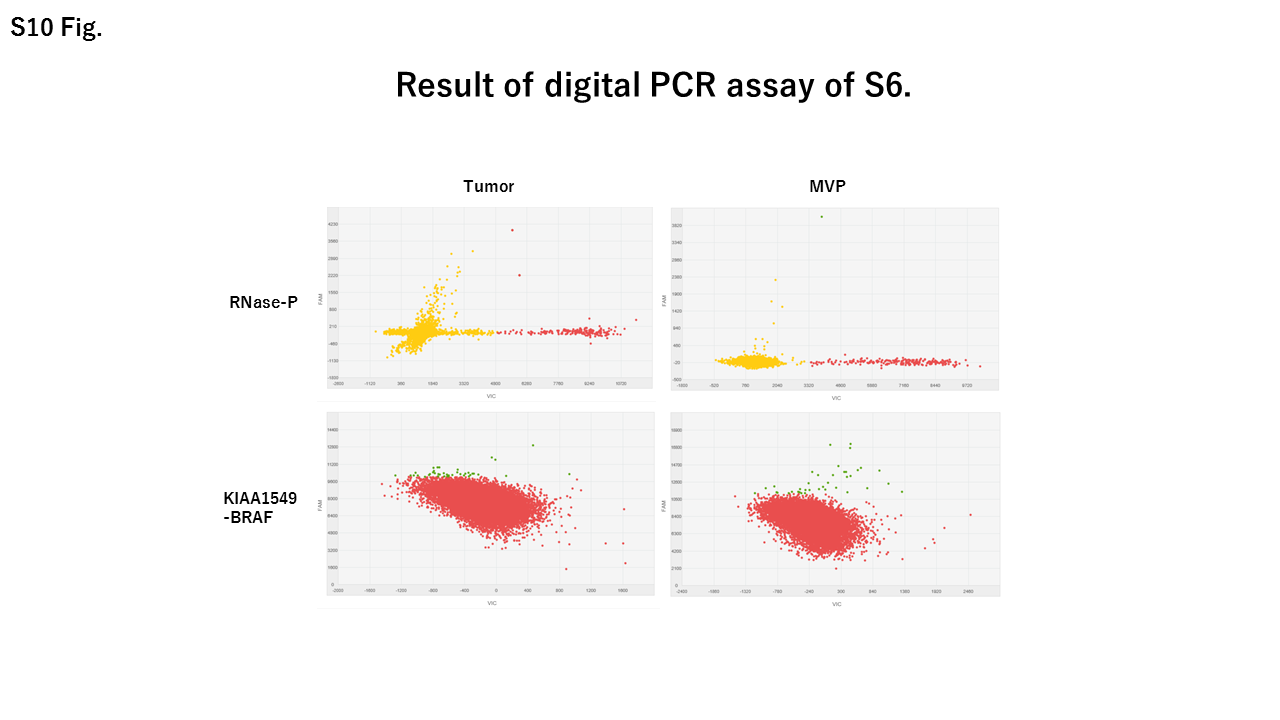

Supplement: S10 Fig — Raw digital PCR data for S6 are shown. Samples from tumor cells and cellular MVP components were independently analyzed. Top: Red and yellow dots indicate droplets with RNase-P DNA and droplets with no DNA, respectively. Bottom: Green and red dots identify droplets with the KIAA-BRAF fusion gene and droplets with no DNA, respectively. (TIF) [file pone.0220146.s010.tif]

## Slide 1
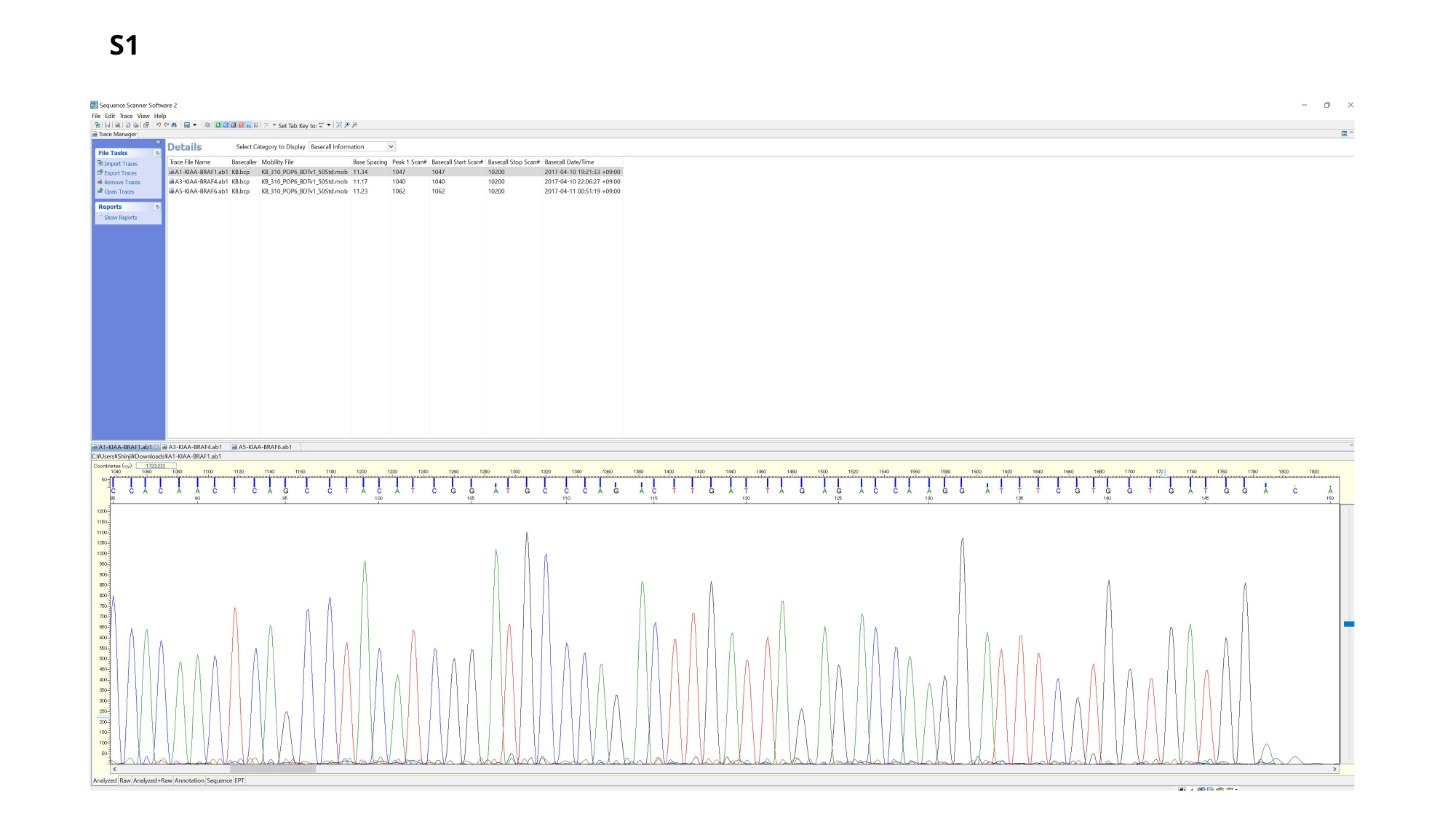

S1

## Slide 2
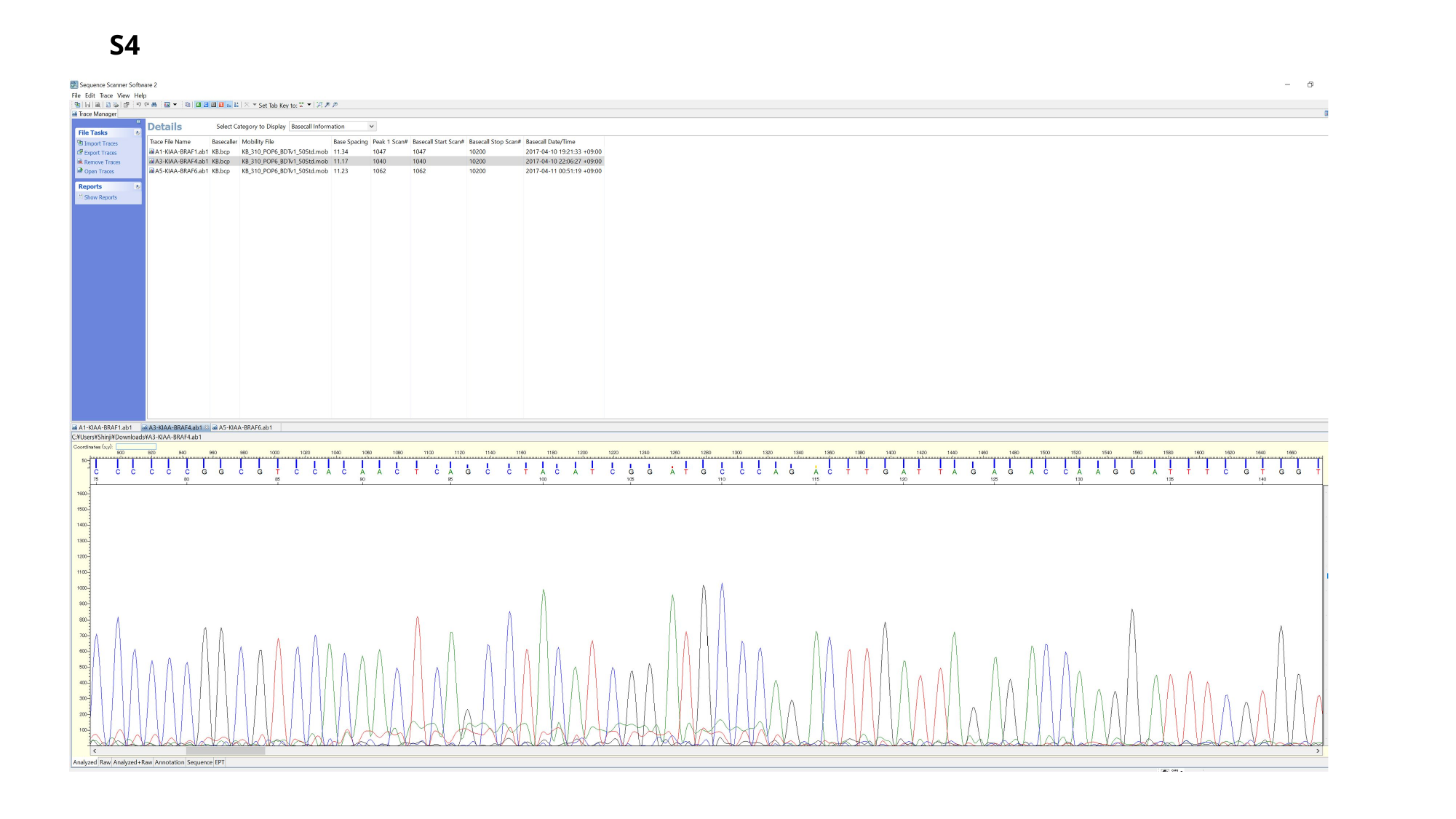

S4

## Slide 3
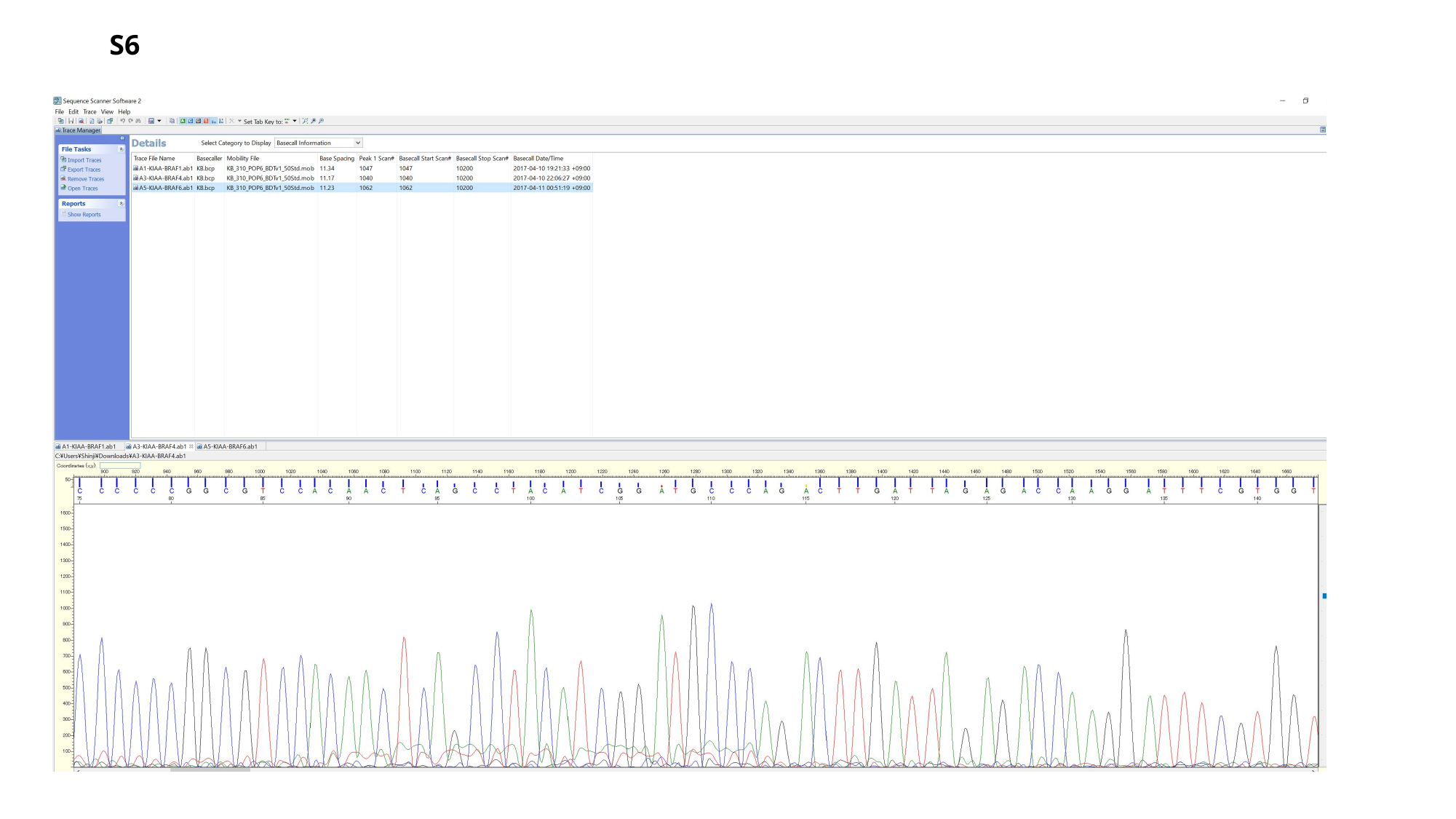

S6

Supplement: S1 Dataset — Raw data of Figs 1B, 3D, 4A, 4B, 5 and S3, S5, S7, S8 and S9 Figs are shown by power point or excel files. Original sequencing data and digital PCR data could be seen with adequate software (Sequence scanner version 2 and QuantStudio 3D Analysis Suite Cloud). These raw data are also available at Dryad digital repository (DOI: https://doi.org/10.5061/dryad.bv44rk5). (ZIP) [file pone.0220146.s011.zip › raw data new/figure 1B/figure 1B raw.pptx]

## Slide 1
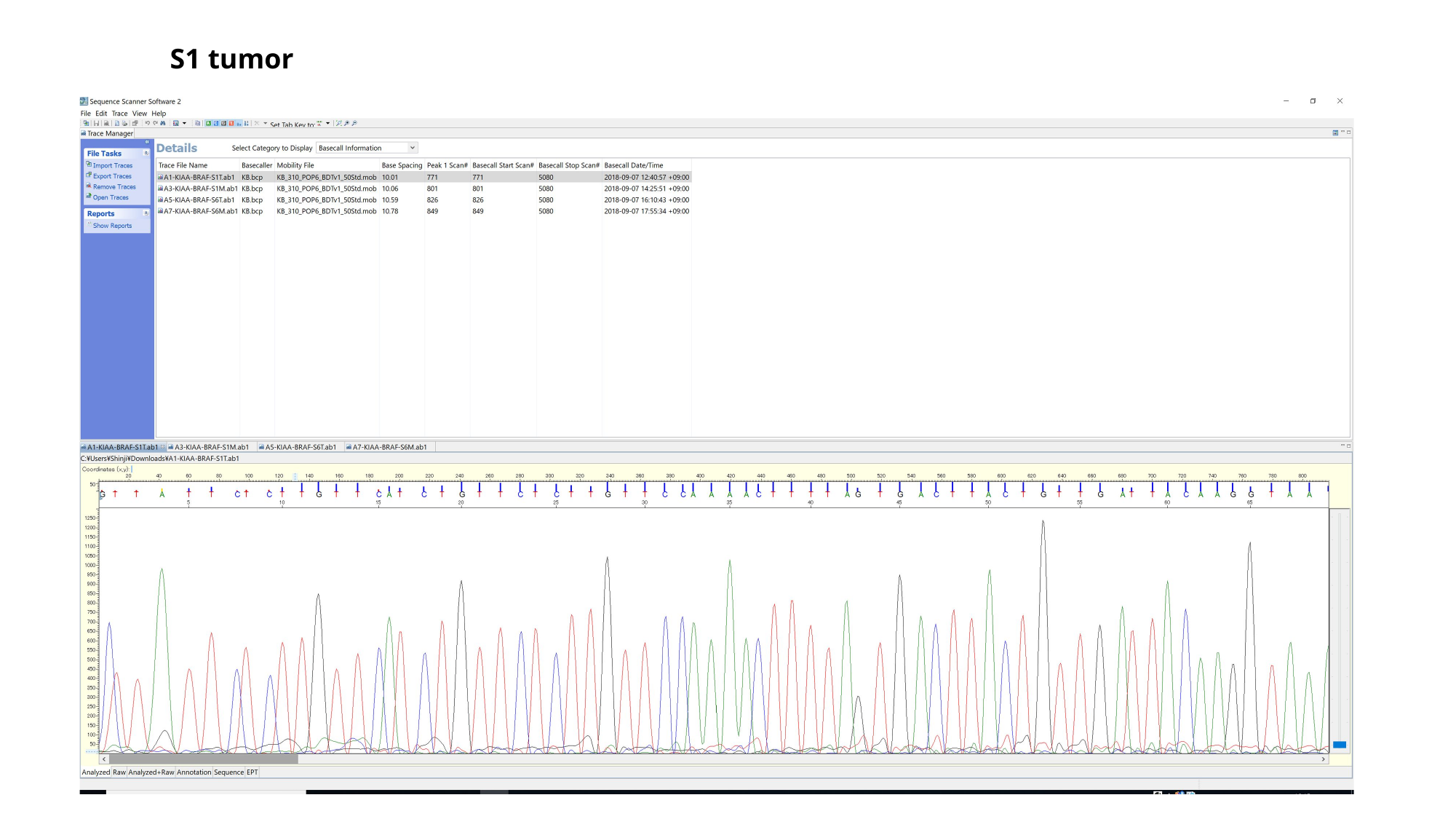

S1 tumor

## Slide 2
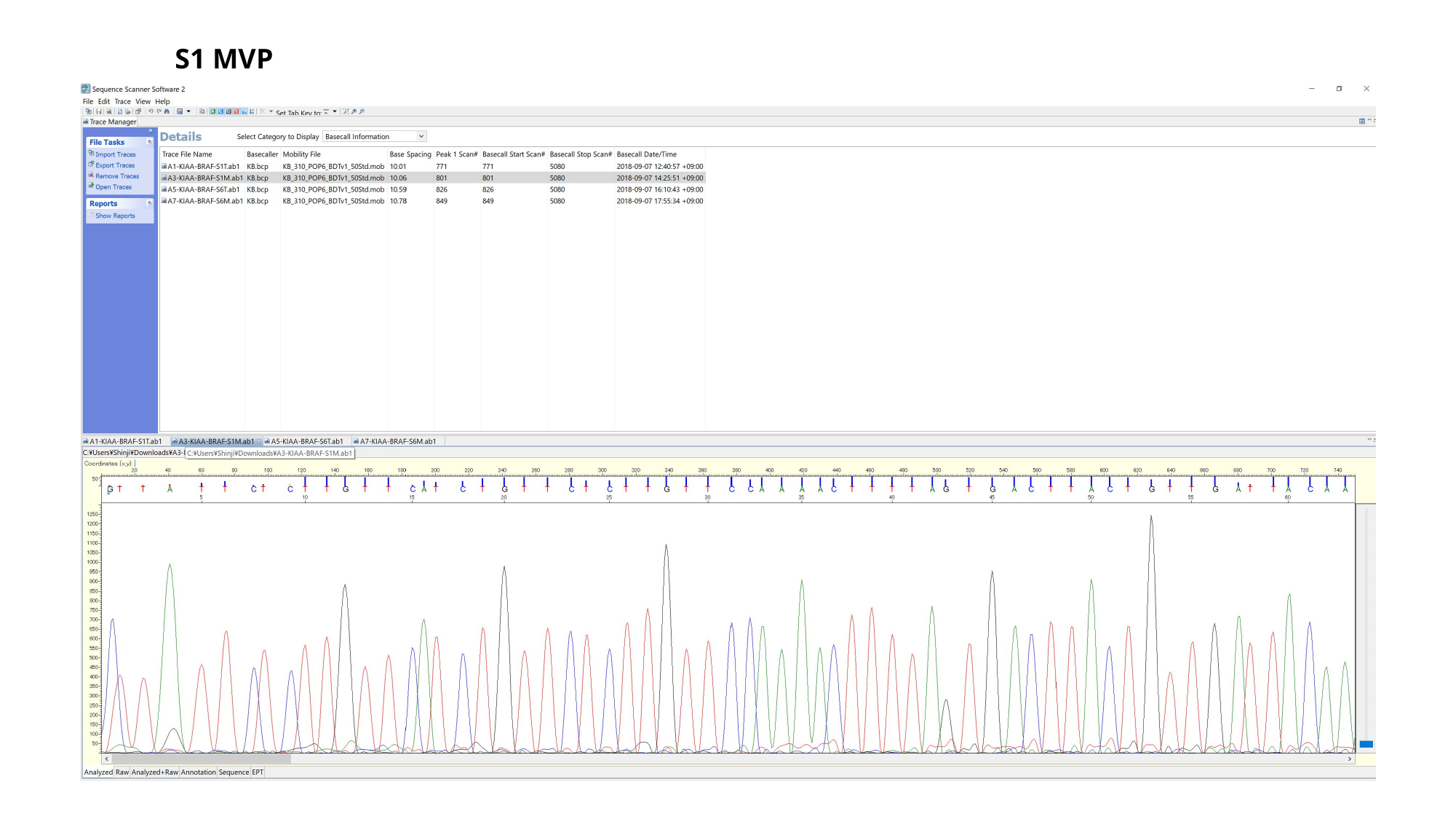

S1 MVP

## Slide 3
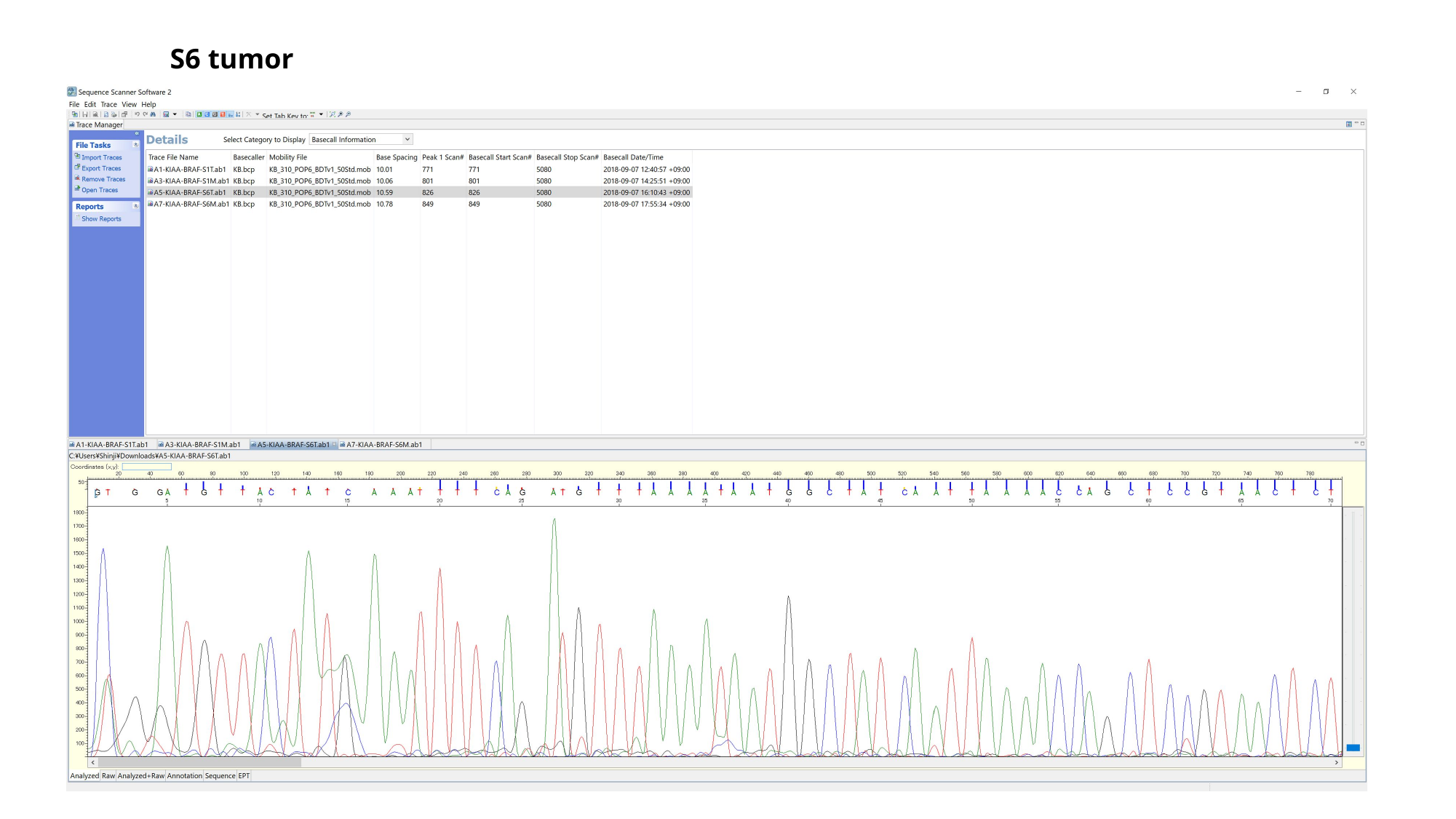

S6 tumor

## Slide 4
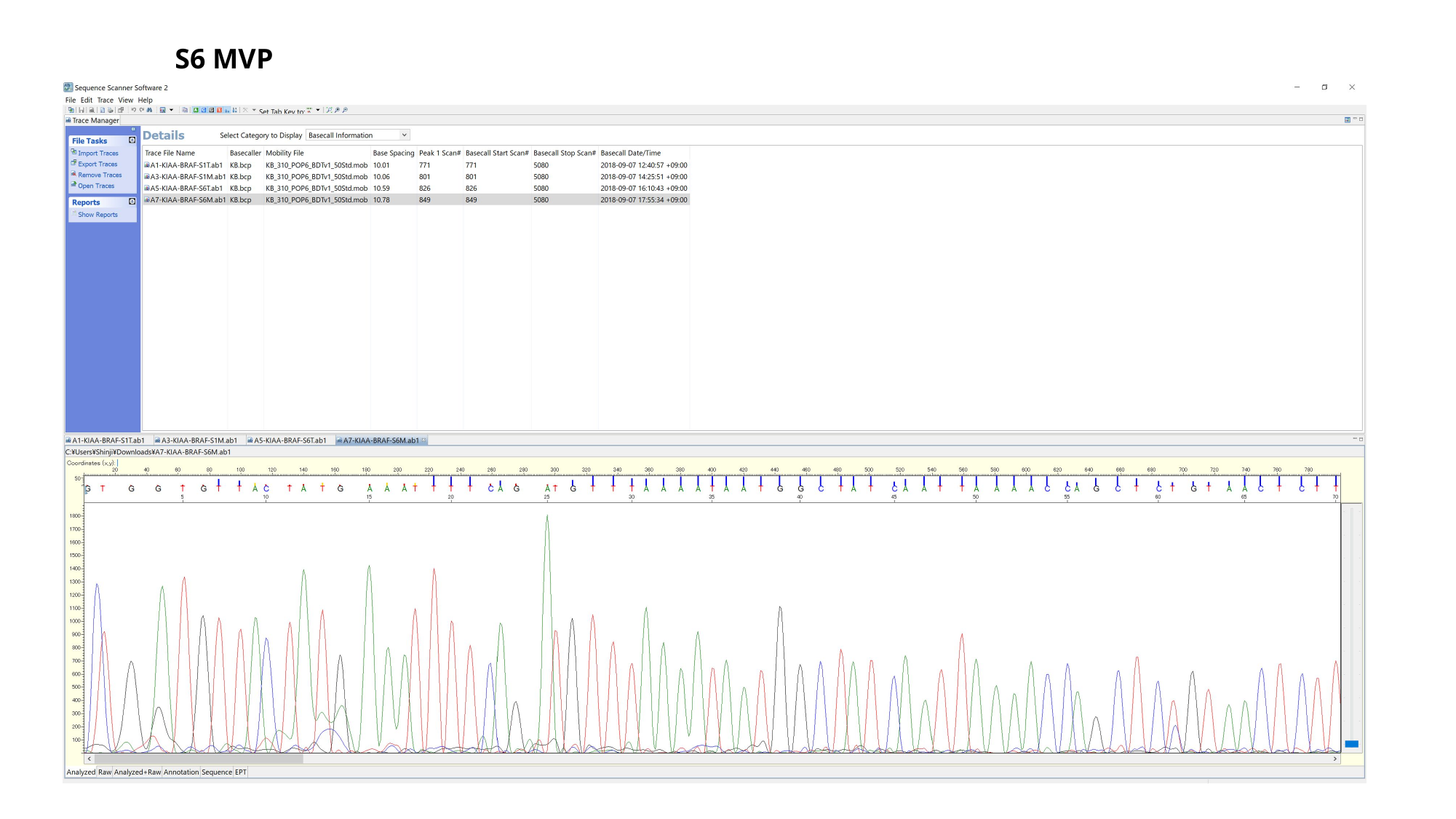

S6 MVP

Supplement: S1 Dataset — Raw data of Figs 1B, 3D, 4A, 4B, 5 and S3, S5, S7, S8 and S9 Figs are shown by power point or excel files. Original sequencing data and digital PCR data could be seen with adequate software (Sequence scanner version 2 and QuantStudio 3D Analysis Suite Cloud). These raw data are also available at Dryad digital repository (DOI: https://doi.org/10.5061/dryad.bv44rk5). (ZIP) [file pone.0220146.s011.zip › raw data new/figure 3D/figure 3D raw.pptx]

## Slide 1
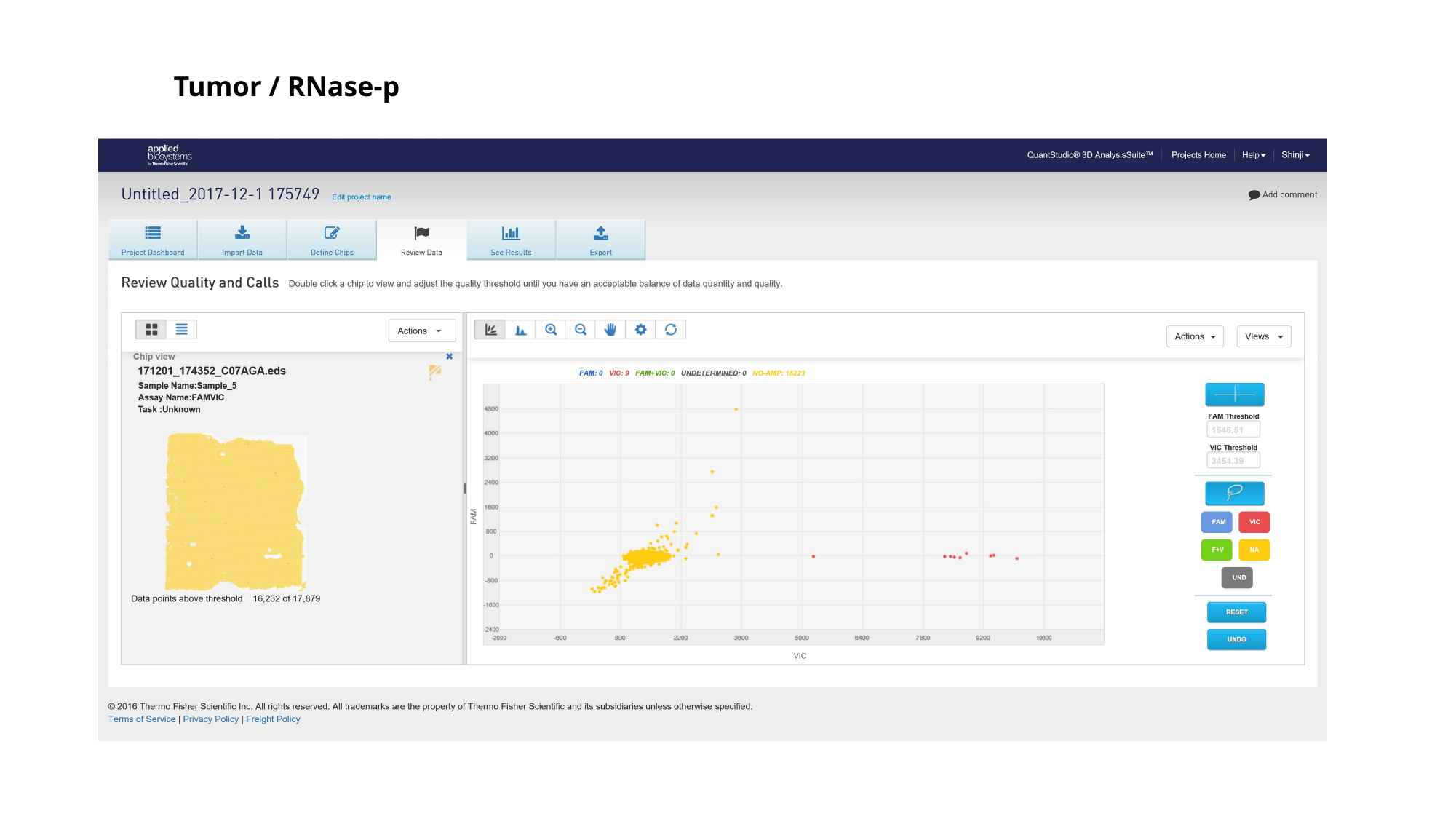

Tumor / RNase-p

## Slide 2
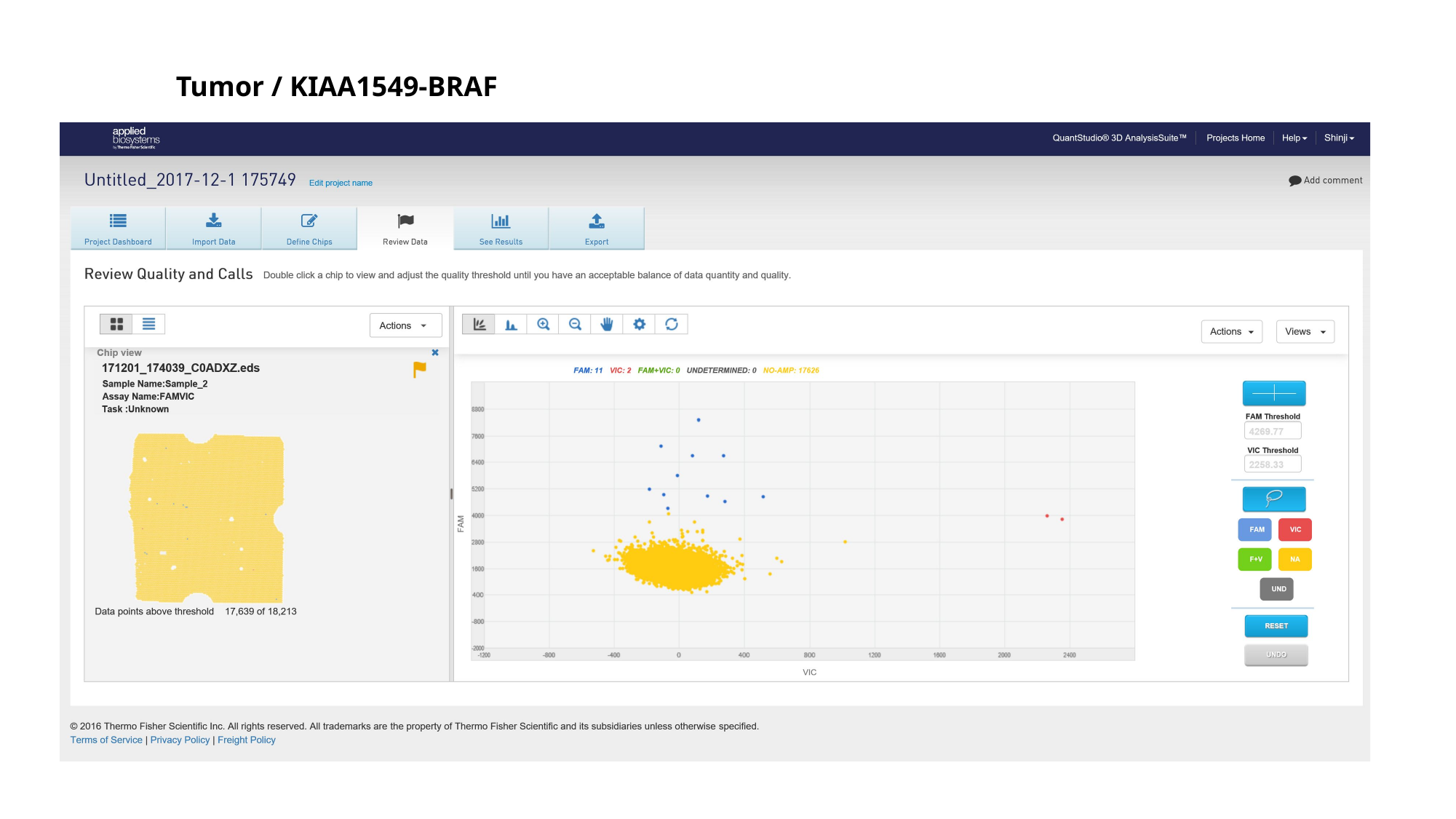

Tumor / KIAA1549-BRAF

## Slide 3
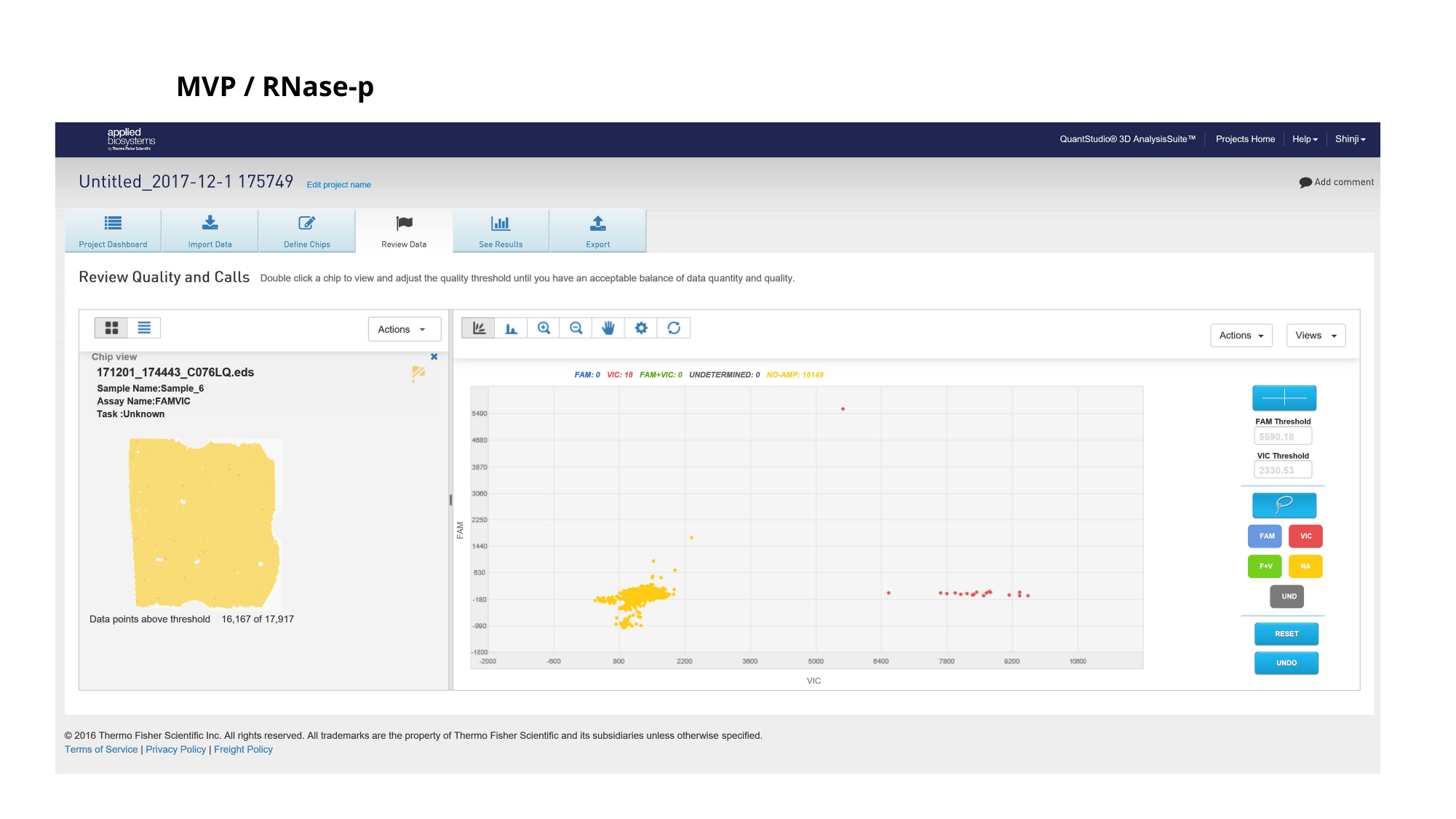

MVP / RNase-p

## Slide 4
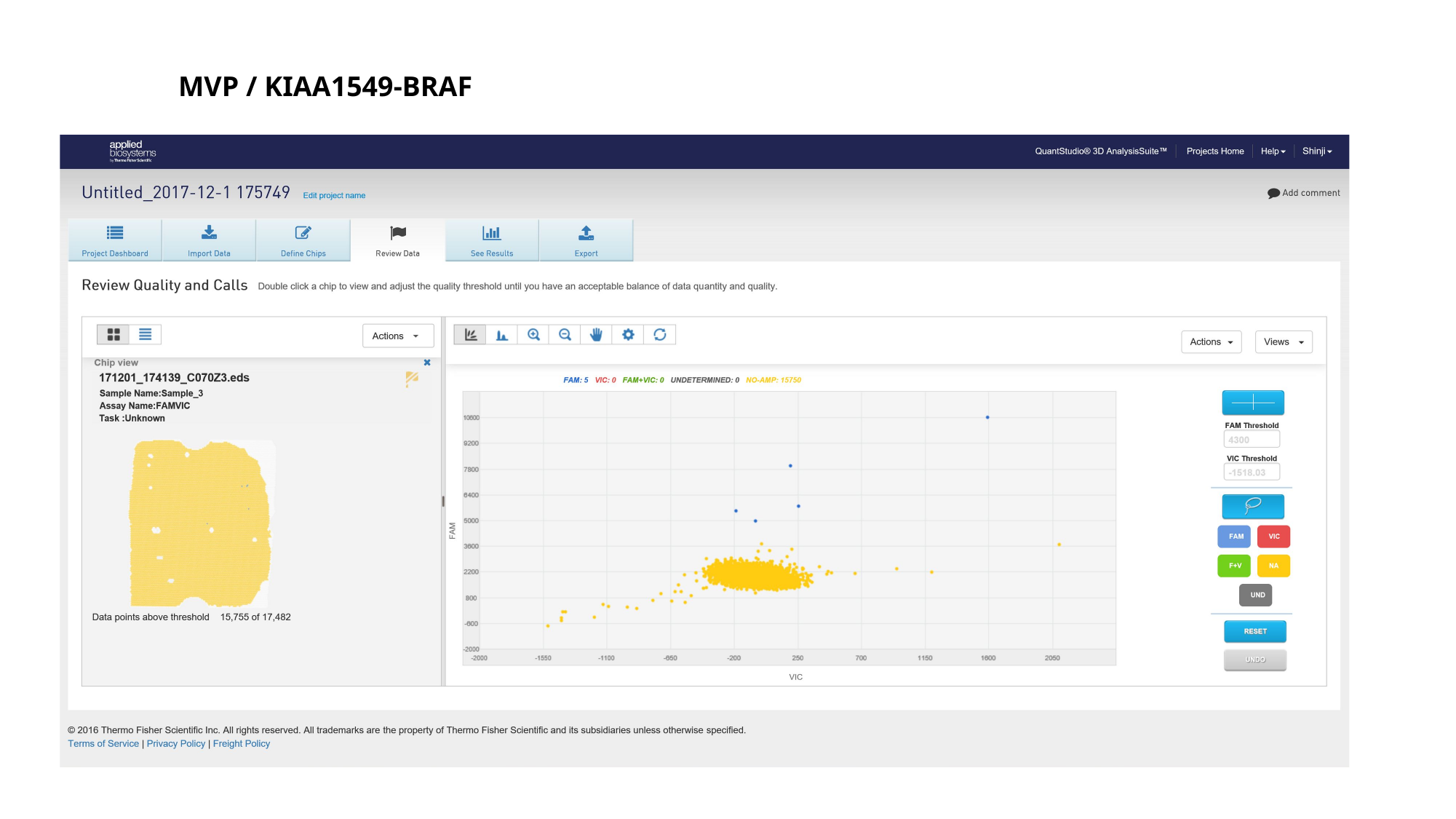

MVP / KIAA1549-BRAF

Supplement: S1 Dataset — Raw data of Figs 1B, 3D, 4A, 4B, 5 and S3, S5, S7, S8 and S9 Figs are shown by power point or excel files. Original sequencing data and digital PCR data could be seen with adequate software (Sequence scanner version 2 and QuantStudio 3D Analysis Suite Cloud). These raw data are also available at Dryad digital repository (DOI: https://doi.org/10.5061/dryad.bv44rk5). (ZIP) [file pone.0220146.s011.zip › raw data new/figure 4A/raw digital PCR figure 4A.pptx]

## Slide 1
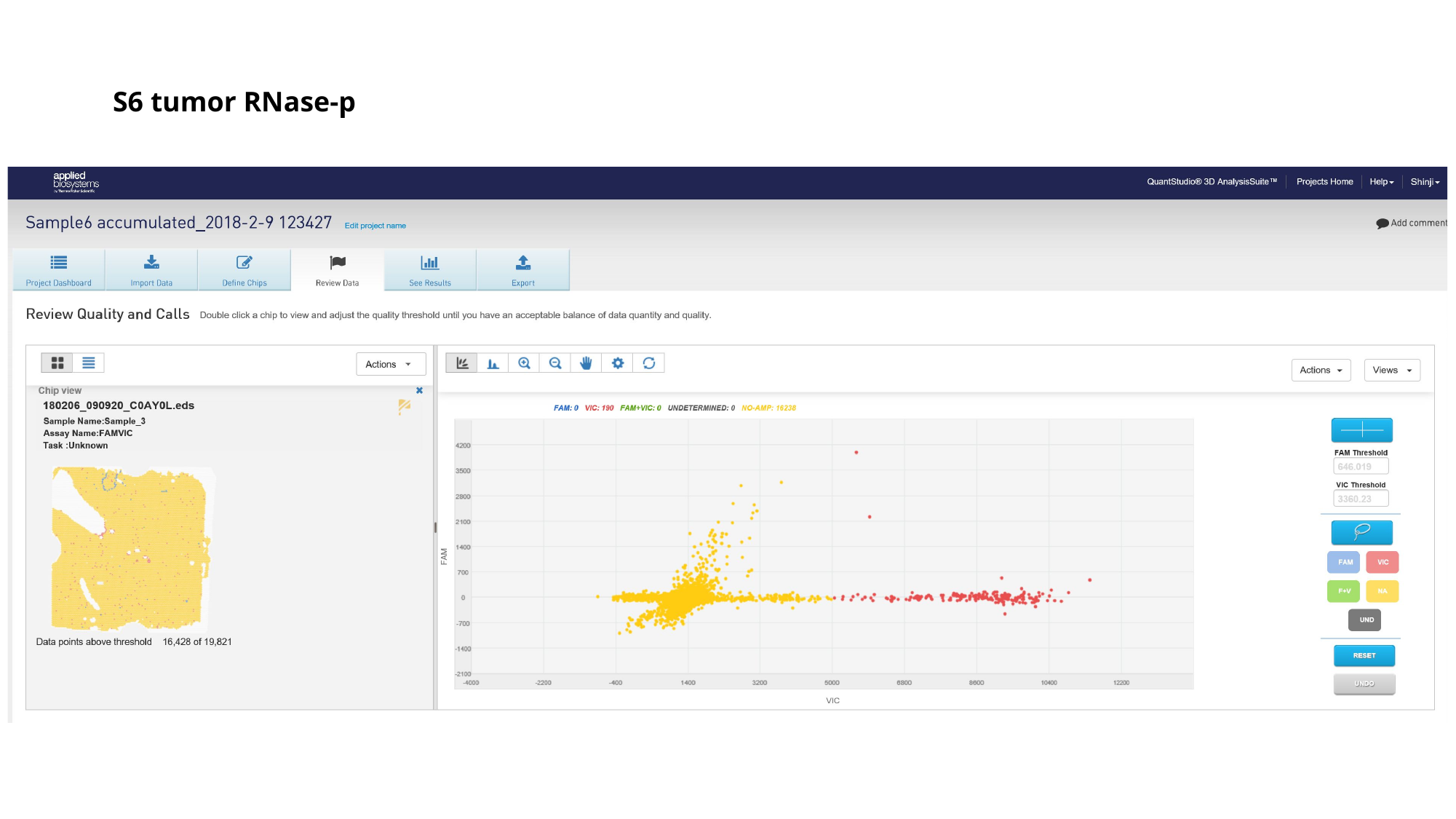

S6 tumor RNase-p

## Slide 2
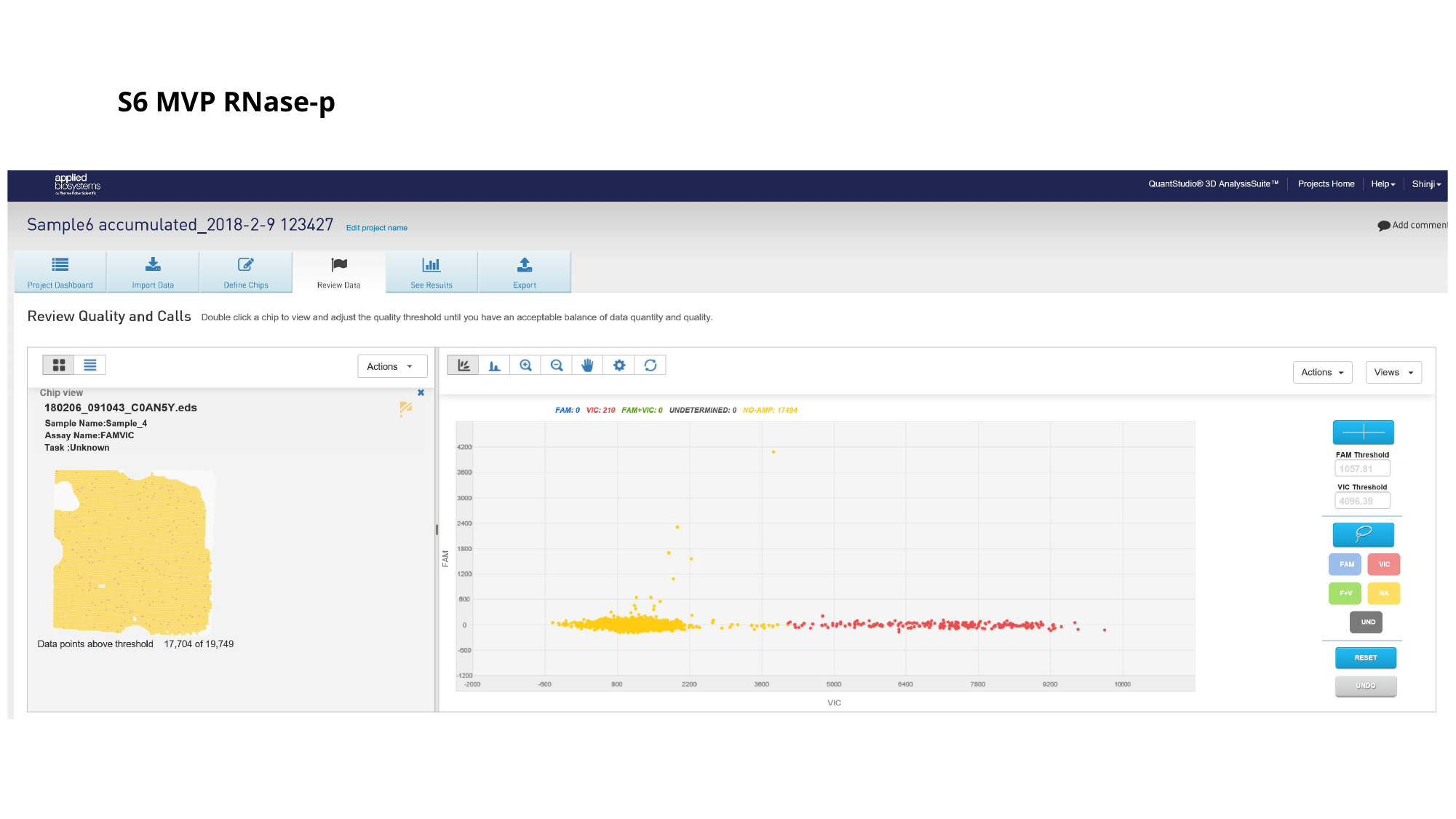

S6 MVP RNase-p

## Slide 3
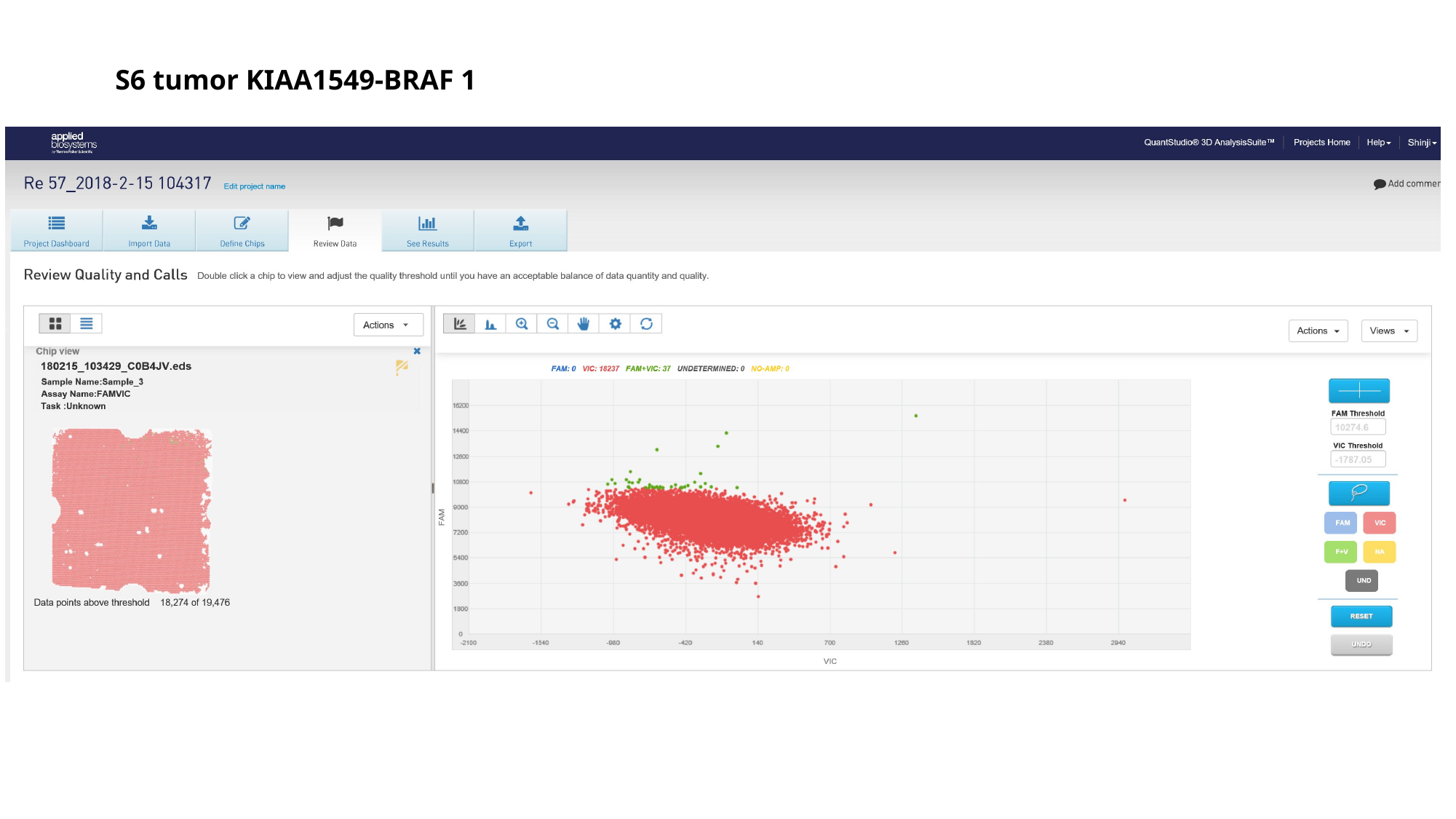

S6 tumor KIAA1549-BRAF 1

## Slide 4
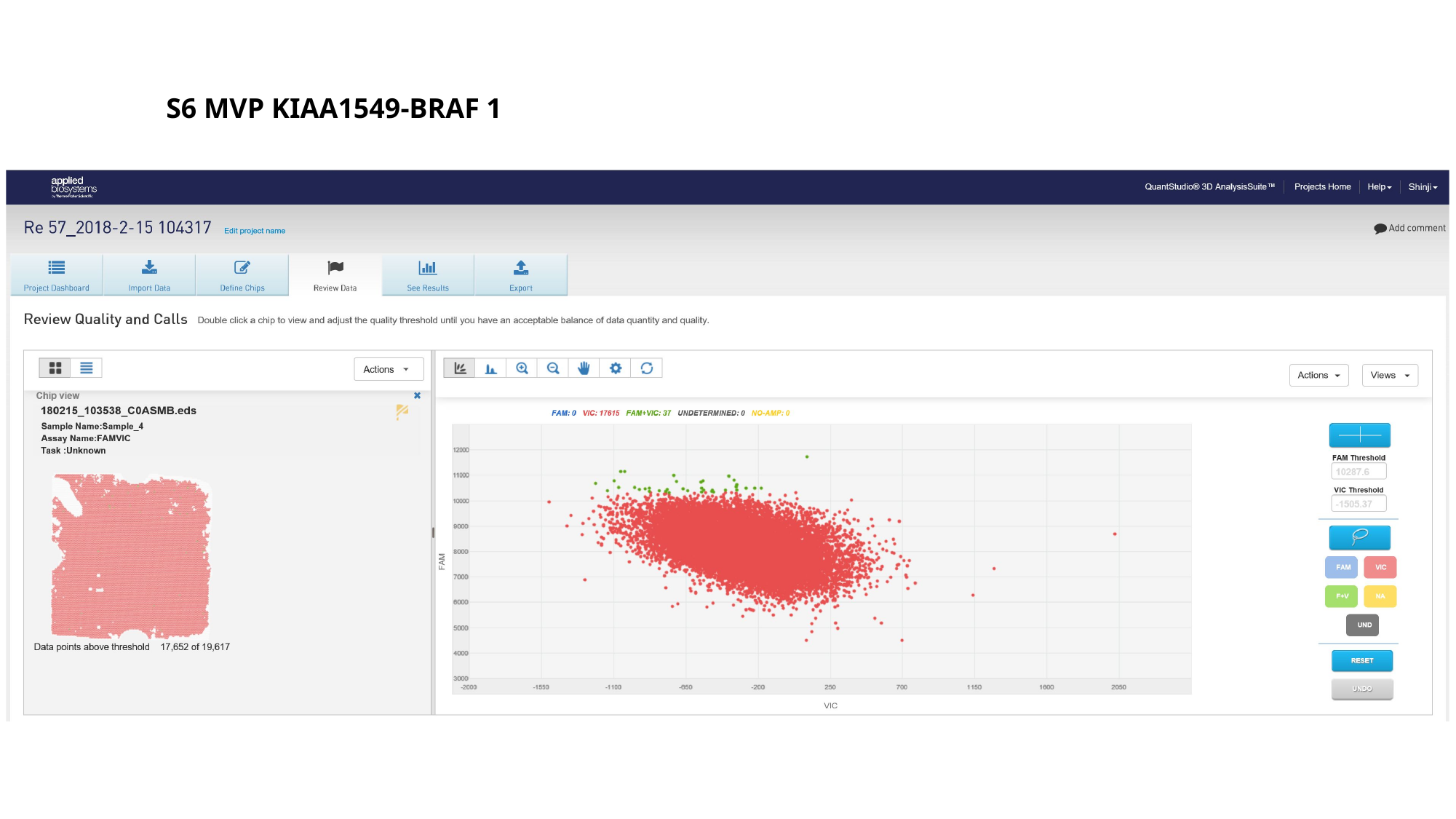

S6 MVP KIAA1549-BRAF 1

Supplement: S1 Dataset — Raw data of Figs 1B, 3D, 4A, 4B, 5 and S3, S5, S7, S8 and S9 Figs are shown by power point or excel files. Original sequencing data and digital PCR data could be seen with adequate software (Sequence scanner version 2 and QuantStudio 3D Analysis Suite Cloud). These raw data are also available at Dryad digital repository (DOI: https://doi.org/10.5061/dryad.bv44rk5). (ZIP) [file pone.0220146.s011.zip › raw data new/figure S9/figure S9 raw data.pptx]
