# Supplementary material for: Detection of the KIAA1549-BRAF fusion gene in cells forming microvascular proliferations in pilocytic astrocytoma
Source: PLoS One. 2019 Jul 22;14(7):e0220146. doi: 10.1371/journal.pone.0220146 (PMC6645544; doi:10.1371/journal.pone.0220146)
Supplement: S1 Dataset — Raw data of Figs 1B, 3D, 4A, 4B, 5 and S3, S5, S7, S8 and S9 Figs are shown by power point or excel files. Original sequencing data and digital PCR data could be seen with adequate software (Sequence scanner version 2 and QuantStudio 3D Analysis Suite Cloud). These raw data are also available at Dryad digital repository (DOI: https://doi.org/10.5061/dryad.bv44rk5). (ZIP) [file pone.0220146.s011.zip › raw data new/figure 4B/S6 digital PCR raw data.pptx]

## Slide 1
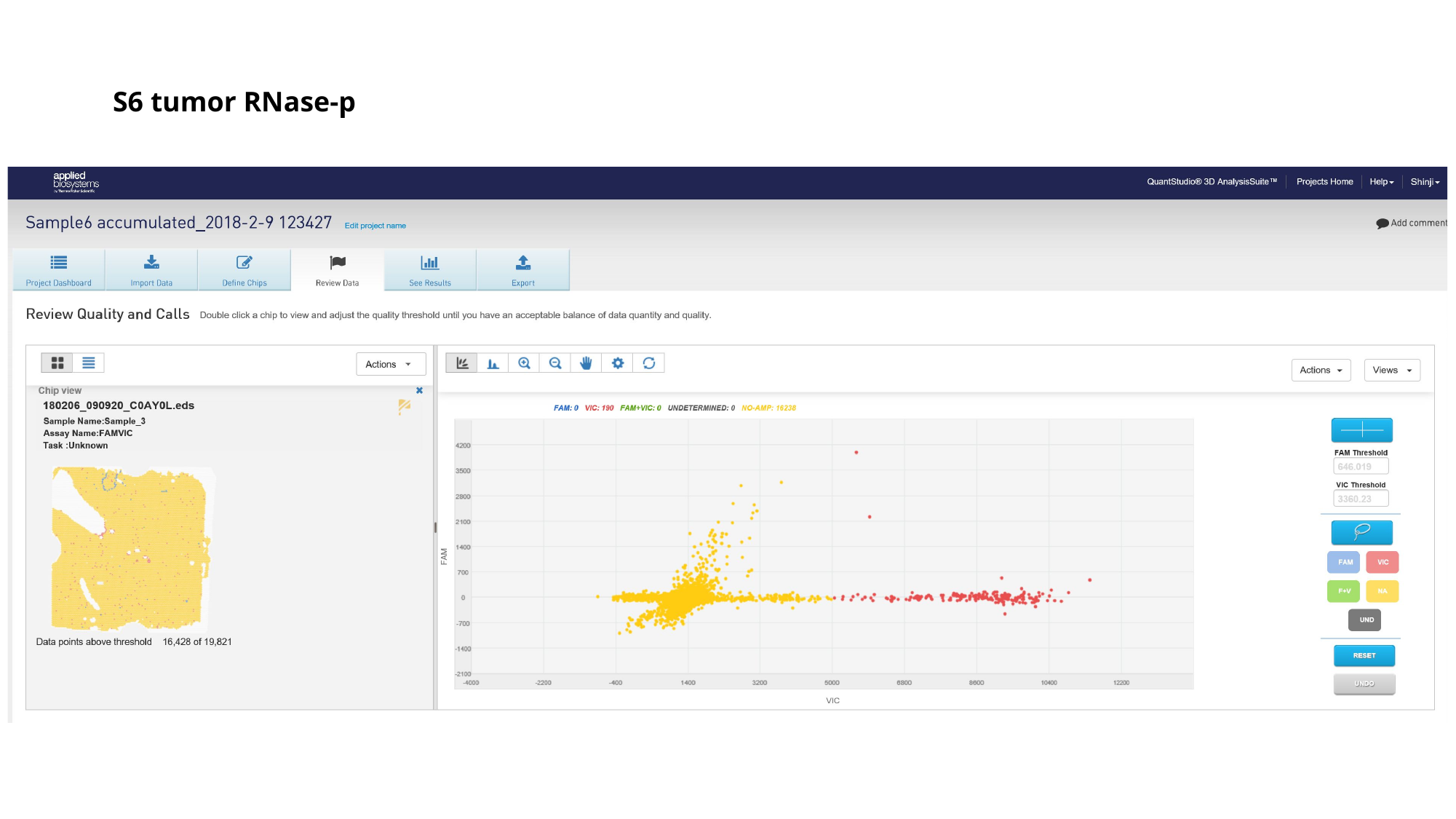

S6 tumor RNase-p

## Slide 2
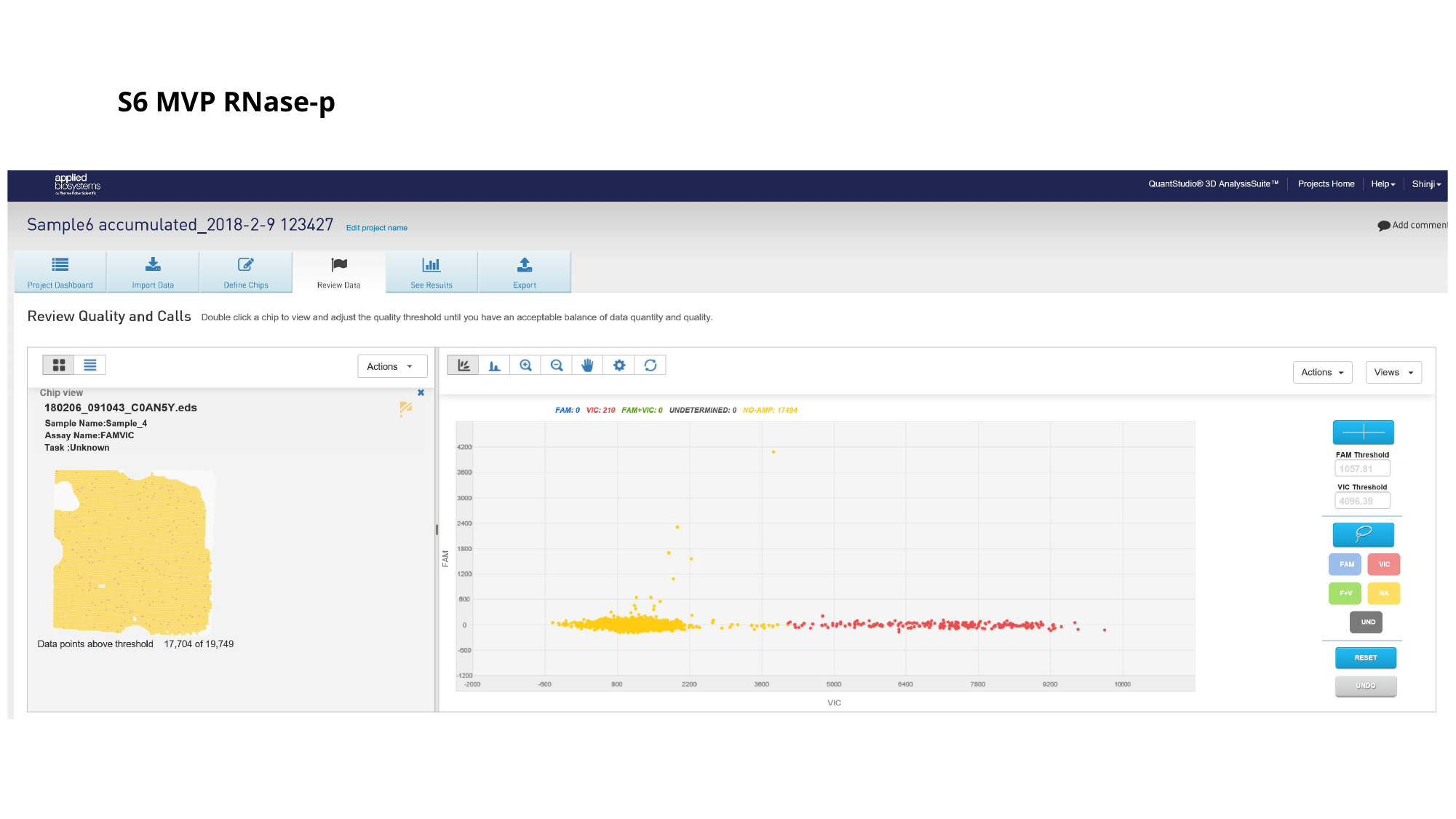

S6 MVP RNase-p

## Slide 3
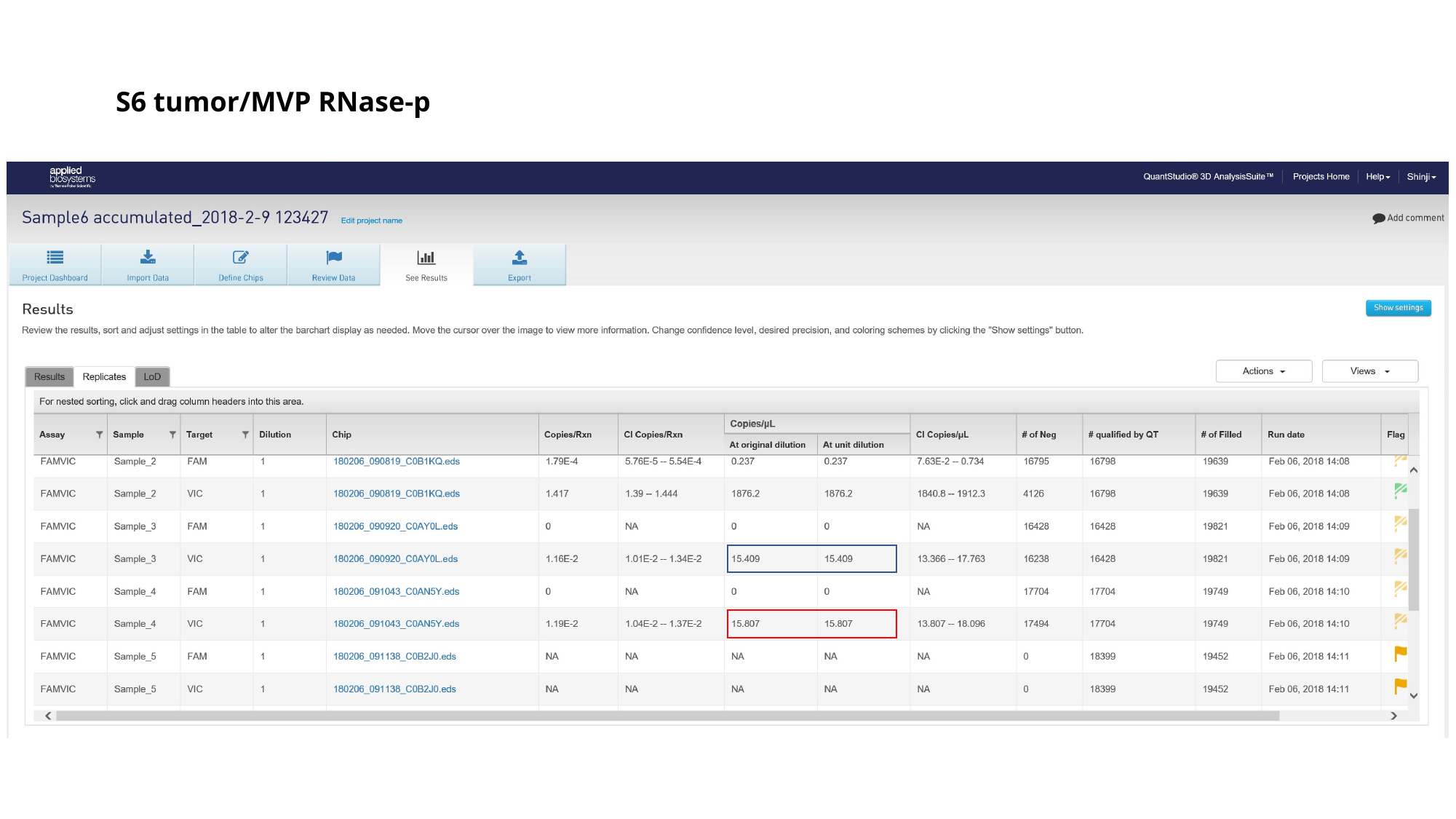

S6 tumor/MVP RNase-p

## Slide 4
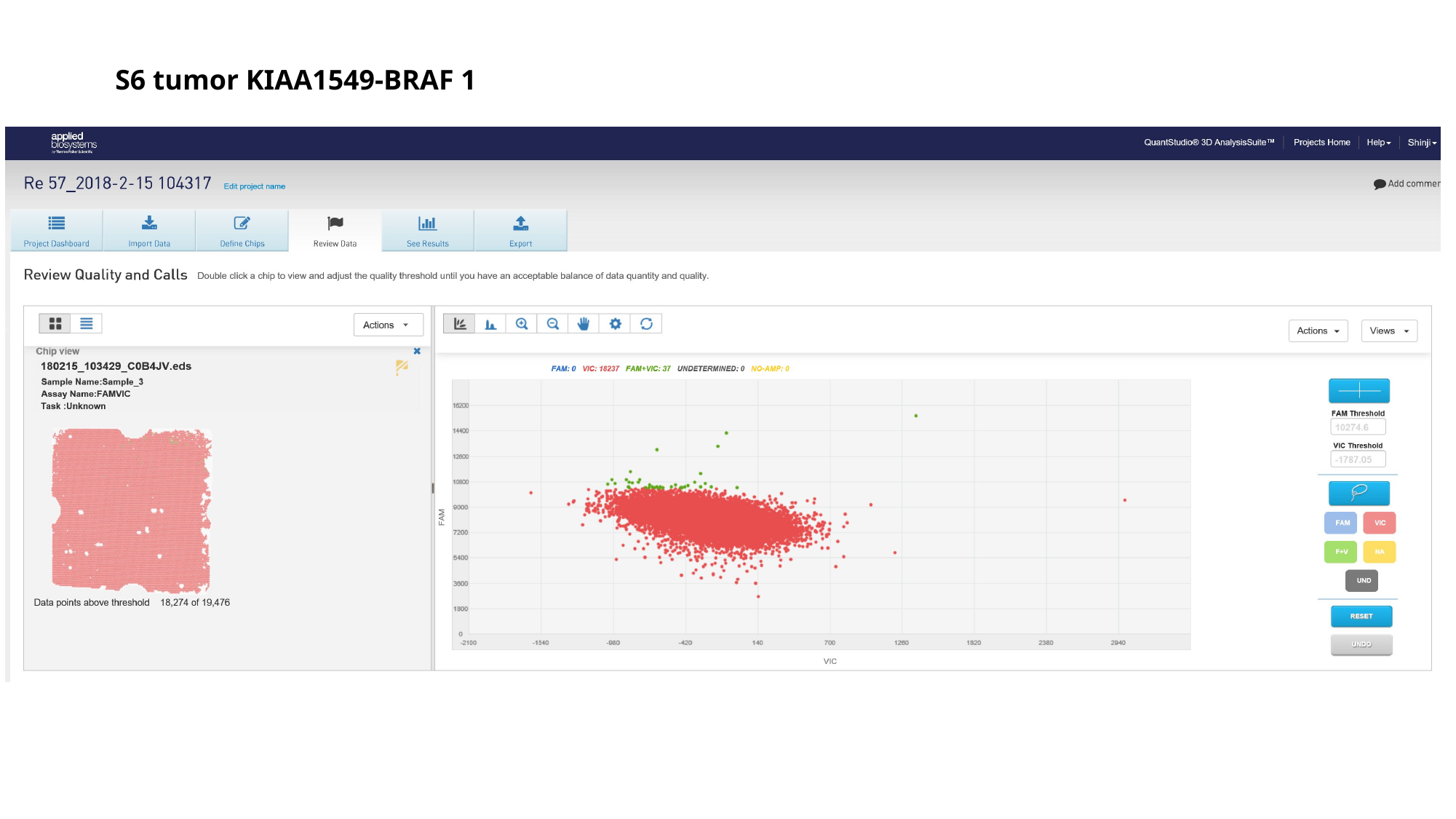

S6 tumor KIAA1549-BRAF 1

## Slide 5
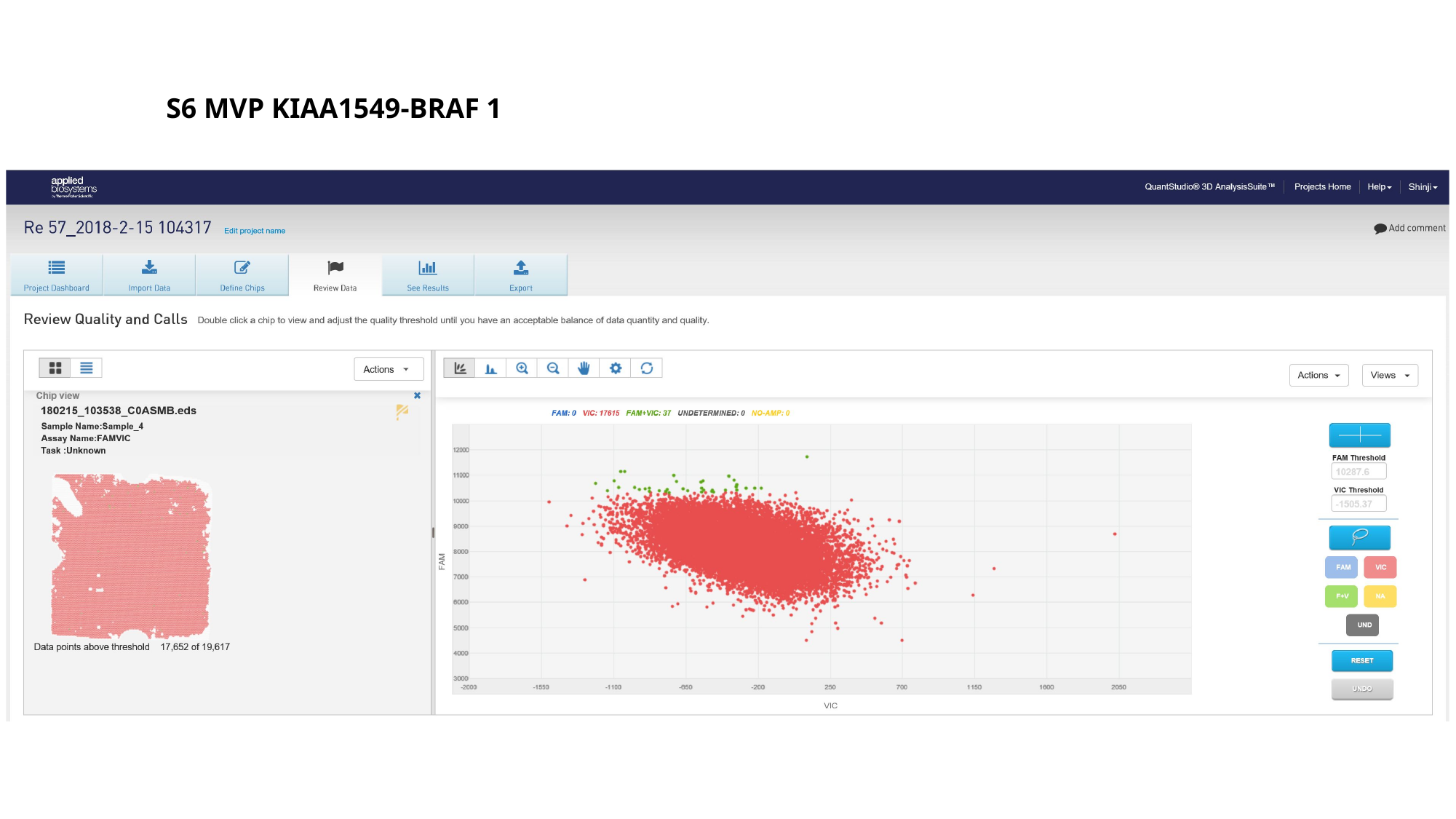

S6 MVP KIAA1549-BRAF 1

## Slide 6
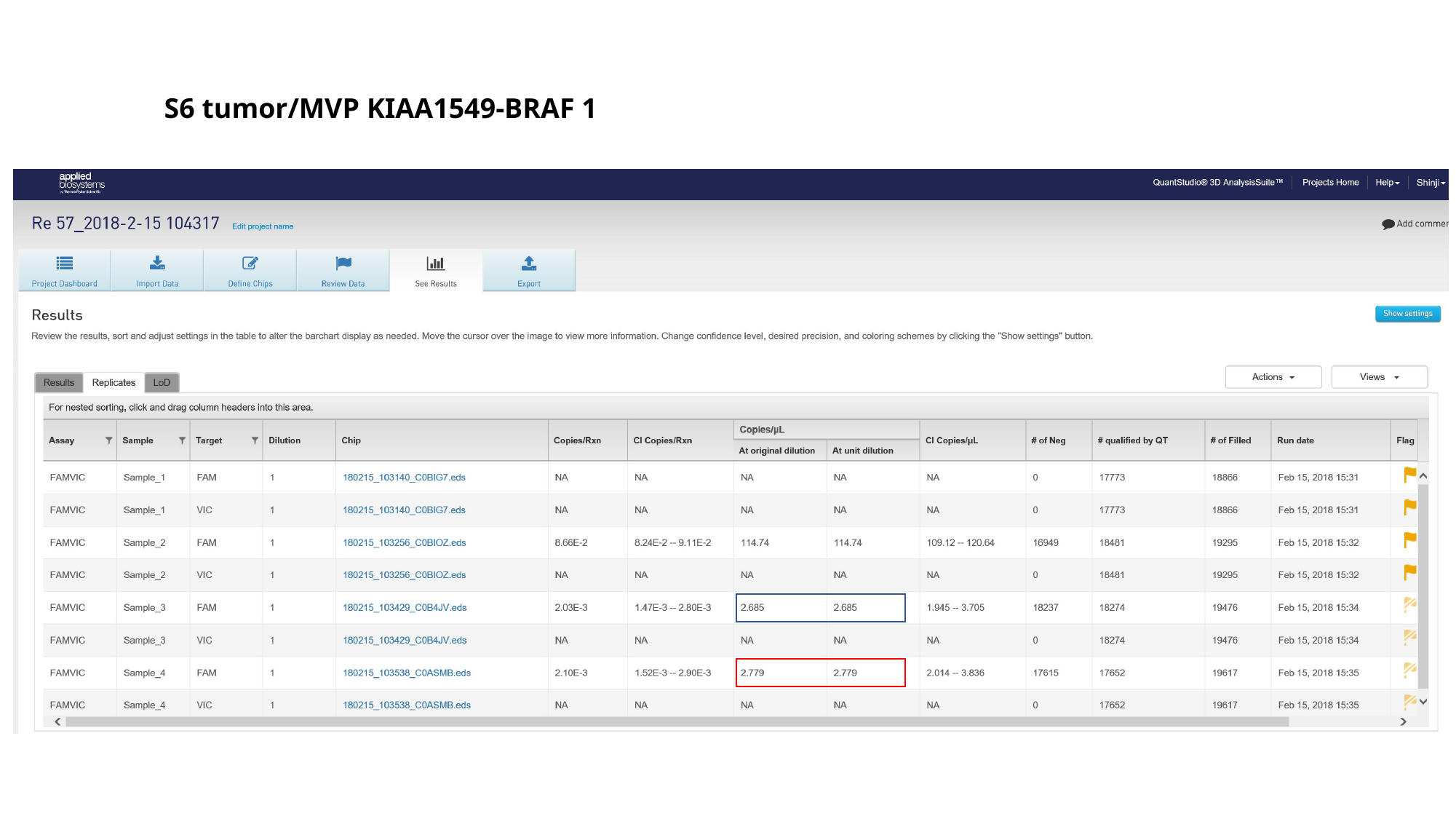

S6 tumor/MVP KIAA1549-BRAF 1

## Slide 7
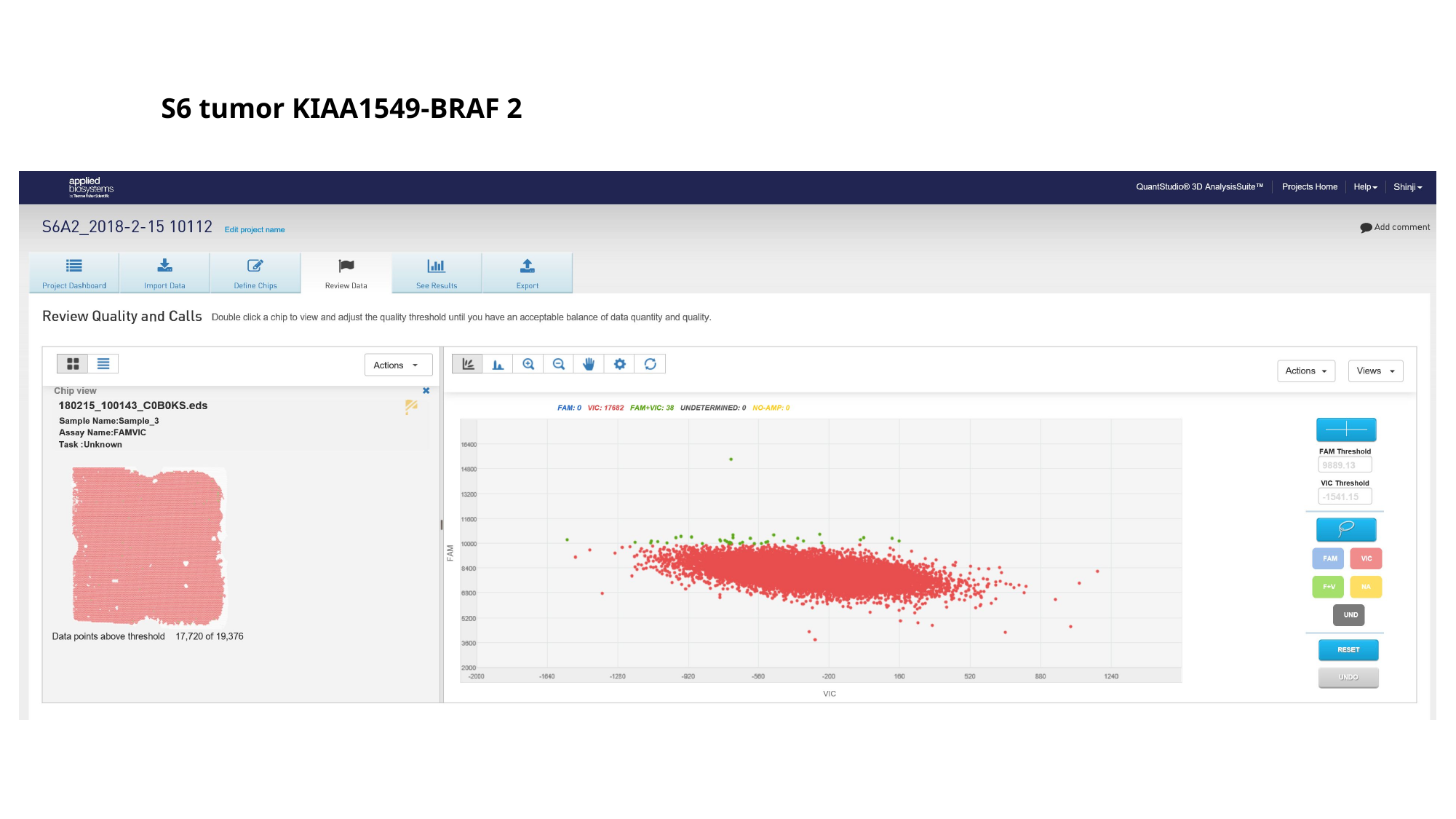

S6 tumor KIAA1549-BRAF 2

## Slide 8
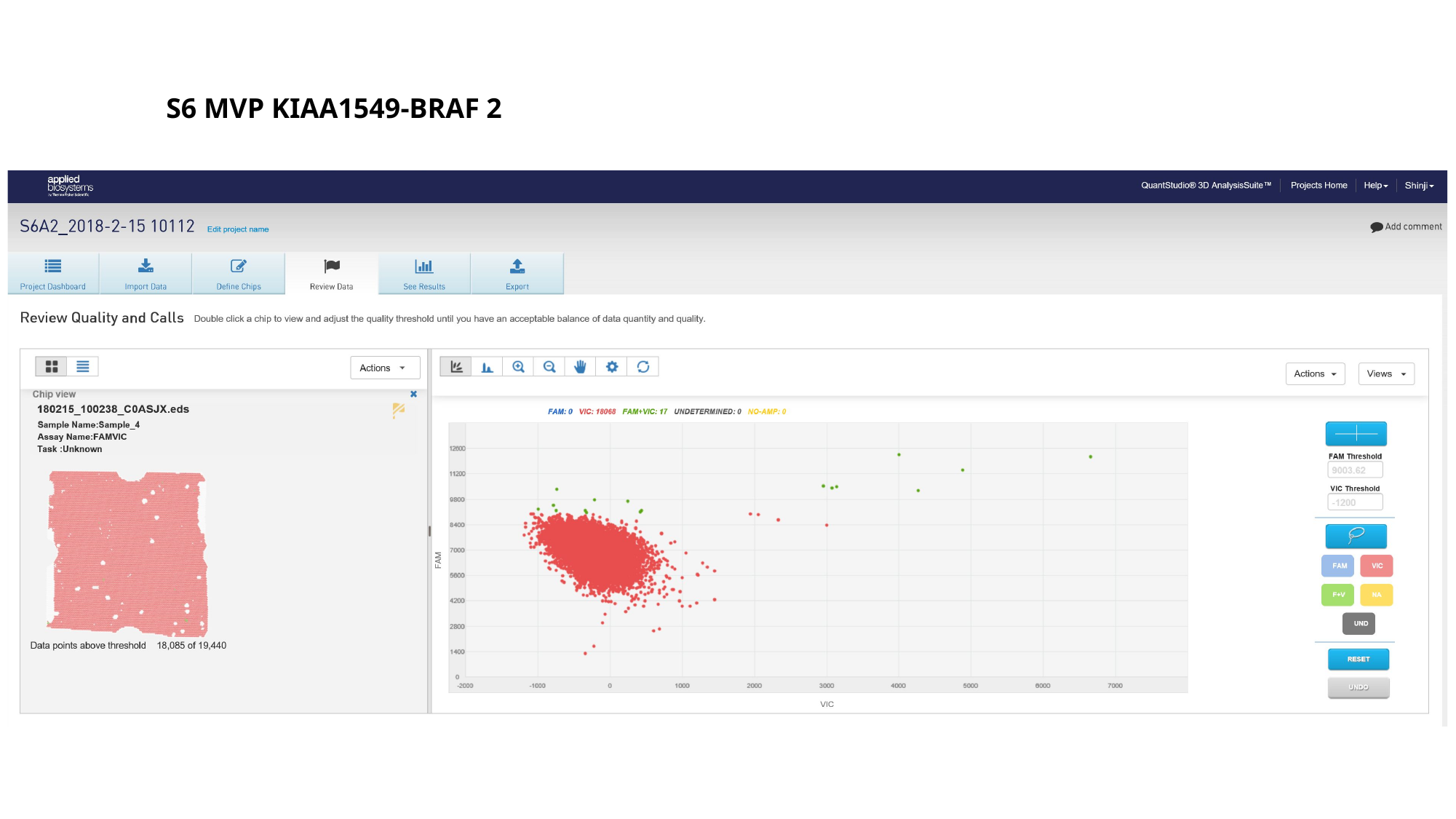

S6 MVP KIAA1549-BRAF 2

## Slide 9
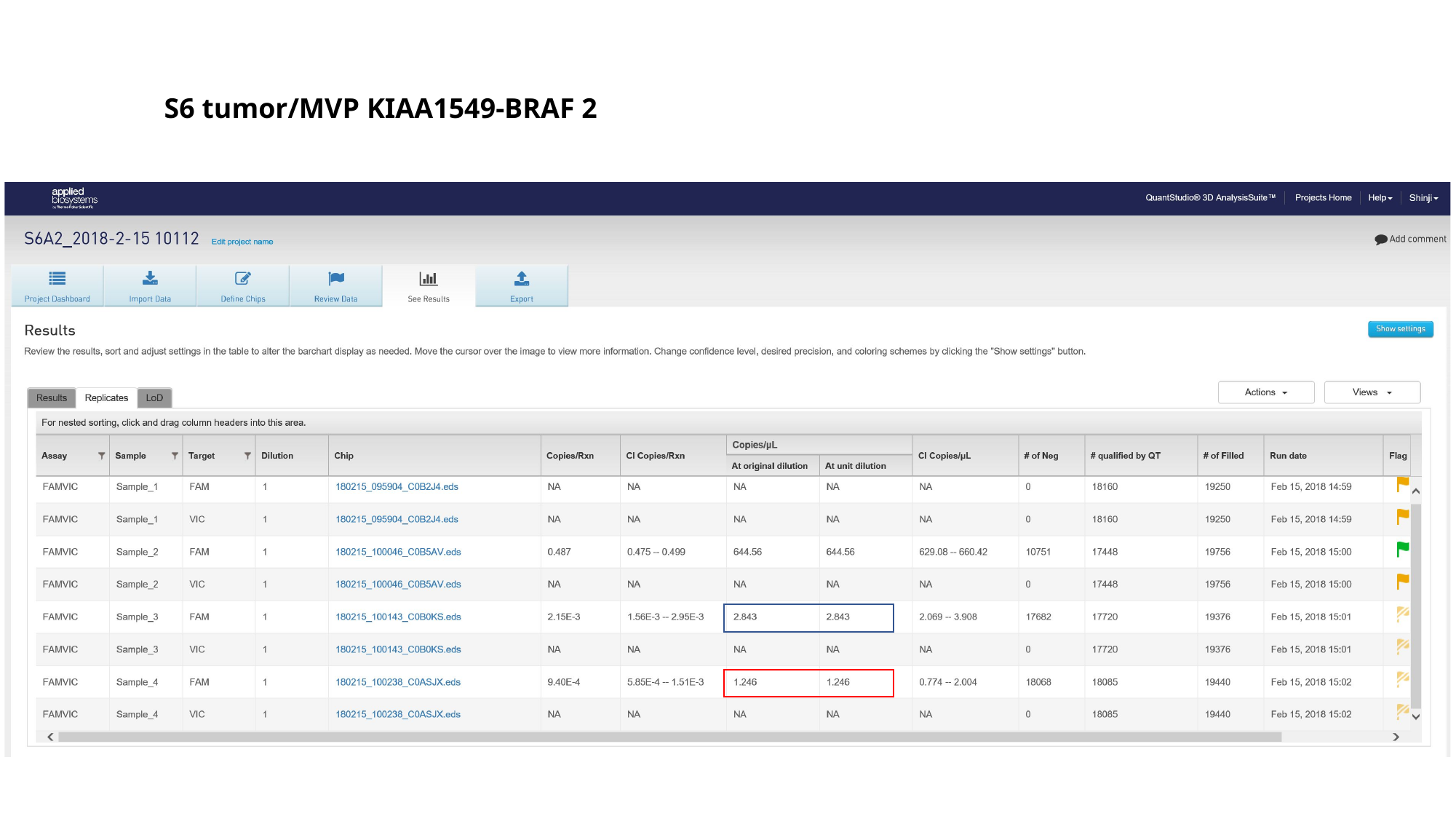

S6 tumor/MVP KIAA1549-BRAF 2

## Slide 10
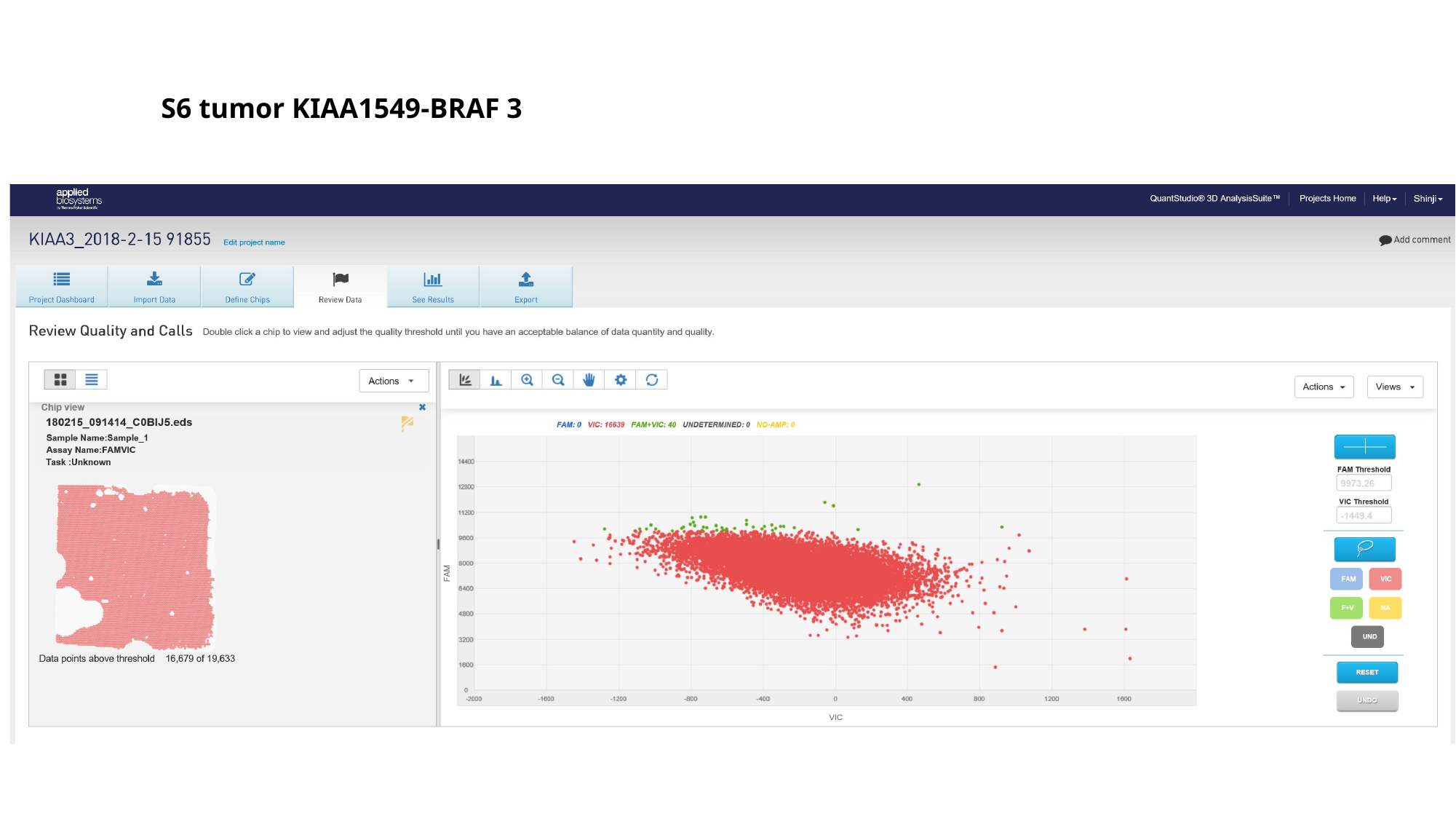

S6 tumor KIAA1549-BRAF 3

## Slide 11
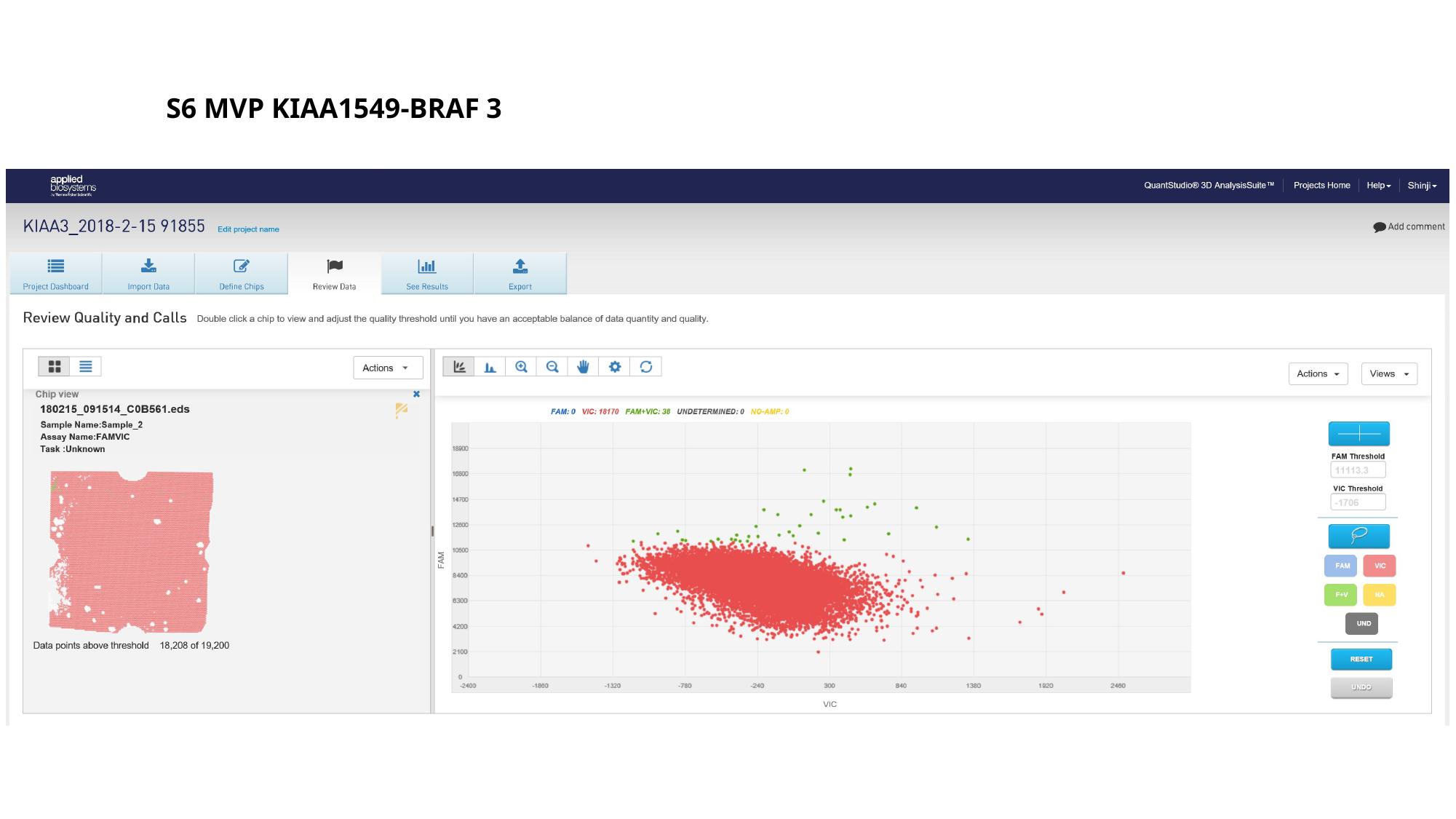

S6 MVP KIAA1549-BRAF 3

## Slide 12
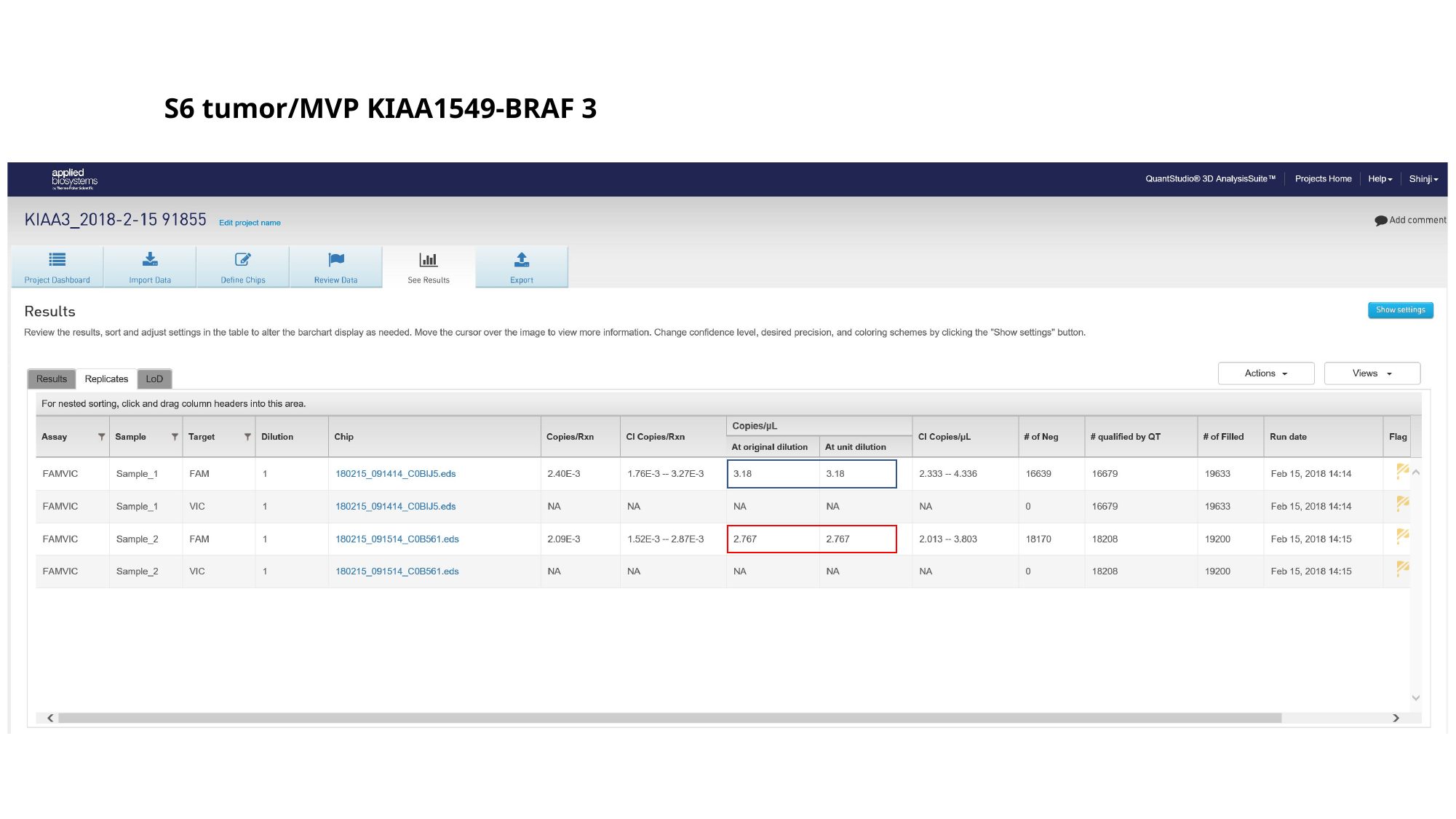

S6 tumor/MVP KIAA1549-BRAF 3

## Slide 13
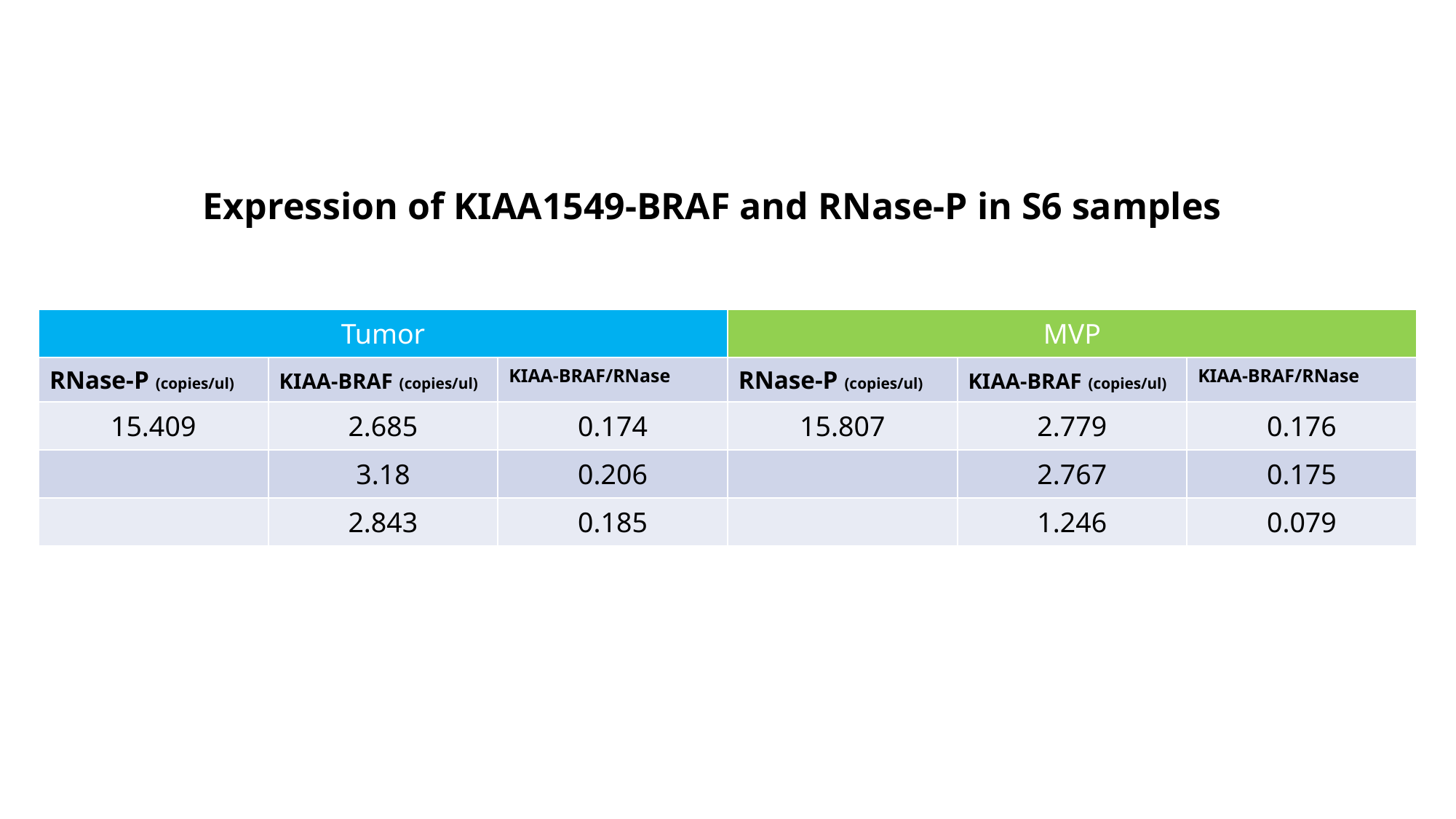

Expression of KIAA1549-BRAF and RNase-P in S6 samples
| Tumor | | | MVP | | |
| --- | --- | --- | --- | --- | --- |
| RNase-P (copies/ul) | KIAA-BRAF (copies/ul) | KIAA-BRAF/RNase | RNase-P (copies/ul) | KIAA-BRAF (copies/ul) | KIAA-BRAF/RNase |
| 15.409 | 2.685 | 0.174 | 15.807 | 2.779 | 0.176 |
| | 3.18 | 0.206 | | 2.767 | 0.175 |
| | 2.843 | 0.185 | | 1.246 | 0.079 |
