# Supplementary material for: Detection of the KIAA1549-BRAF fusion gene in cells forming microvascular proliferations in pilocytic astrocytoma
Source: PLoS One. 2019 Jul 22;14(7):e0220146. doi: 10.1371/journal.pone.0220146 (PMC6645544; doi:10.1371/journal.pone.0220146)
Supplement: S1 Dataset — Raw data of Figs 1B, 3D, 4A, 4B, 5 and S3, S5, S7, S8 and S9 Figs are shown by power point or excel files. Original sequencing data and digital PCR data could be seen with adequate software (Sequence scanner version 2 and QuantStudio 3D Analysis Suite Cloud). These raw data are also available at Dryad digital repository (DOI: https://doi.org/10.5061/dryad.bv44rk5). (ZIP) [file pone.0220146.s011.zip › raw data new/figure 5/figure 5.pptx]

## Slide 1
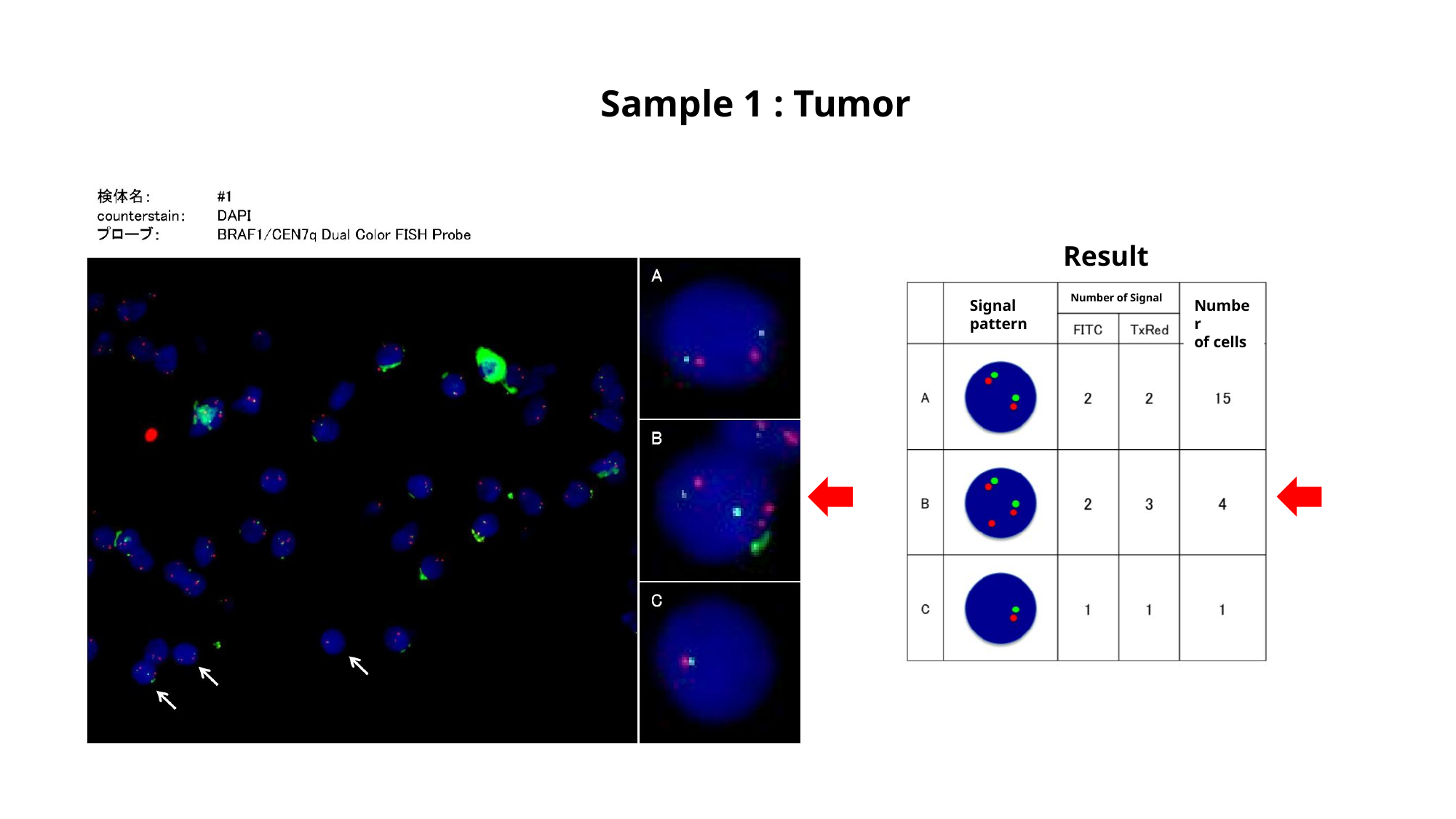

Sample 1 : Tumor
Result
Number of Signal
Signal pattern
Number
of cells

## Slide 2
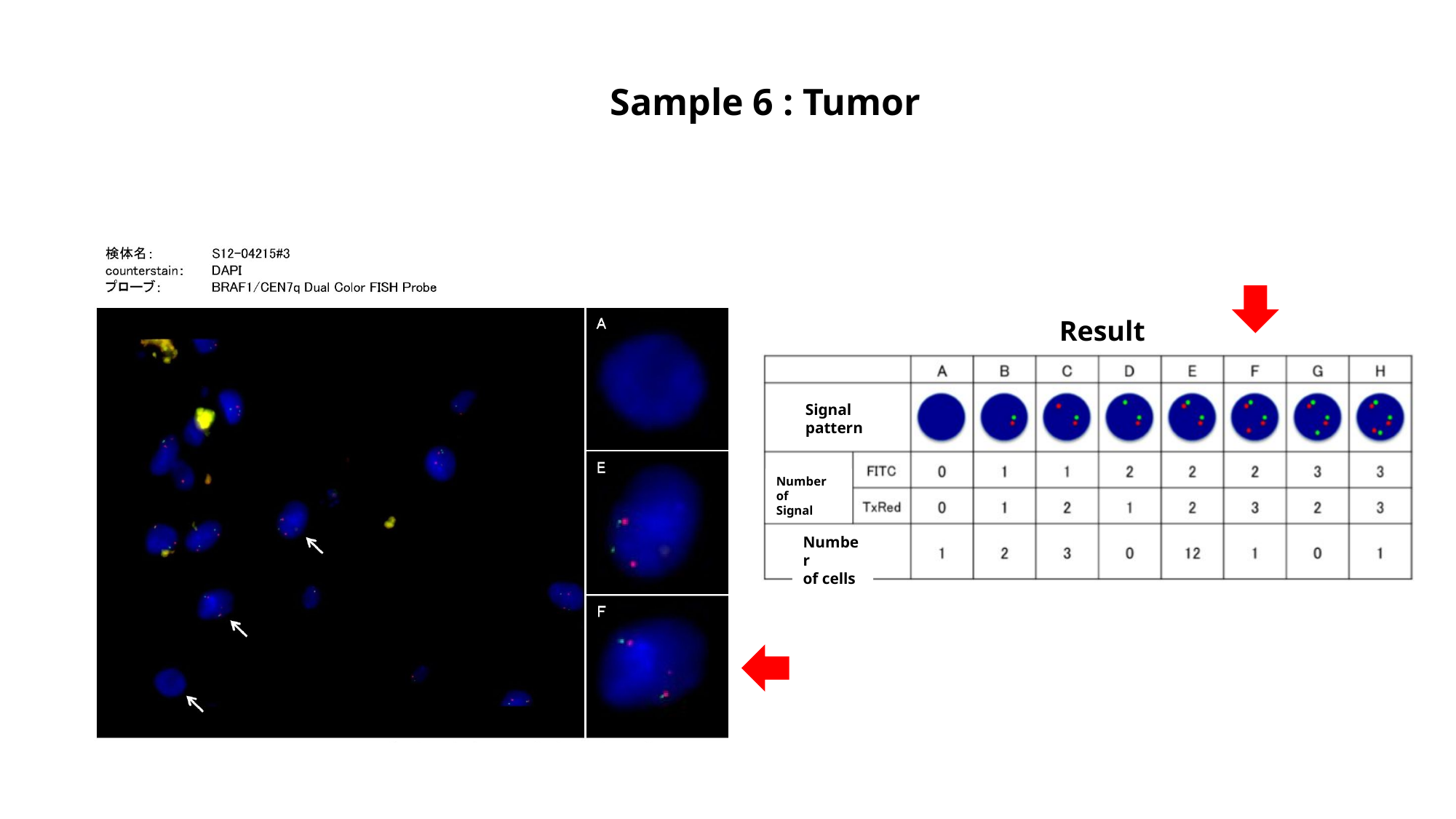

Sample 6 : Tumor
Result
Signal pattern
Number of
Signal
Number
of cells
